# Supplementary material for: How far back do we need to look to capture diagnoses in electronic health records? A retrospective observational study of hospital electronic health record data
Source: BMJ Open. 2024 Feb 13;14(2):e080678. doi: 10.1136/bmjopen-2023-080678 (PMC10868273; doi:10.1136/bmjopen-2023-080678)
Supplement: Supplementary data [file bmjopen-2023-080678supp001.pdf]

Supplementary Information

ADMISSION Research Collaborative Consortium members:

Avan Aihie Sayer[4,5]

Victoria Bartle[8]

Rachel Cooper[4,5]

Heather J Cordell[9]

Ray Holding[8]

Tom Marshall[10]

Fiona E Matthews[11]

Paolo Missier[12]

Ewan Pearson[13]

Chris Plummer[2,5]

Sian Robinson[4,5]

Elizabeth Sapey[1,6]

Mervyn Singer[7]

Thomas Scharf[11]

James Wason[14]

Miles D Witham[4,5]

1. PIONEER Hub, University of Birmingham, Birmingham, UK and Health Informatics, University Hospitals Birmingham NHS Foundation Trust, Birmingham, UK

2. Digital Services, Newcastle upon Tyne Hospitals NHS Foundation Trust, Newcastle upon Tyne, UK

4. AGE Research Group, Translational and Clinical Research Institute, Faculty of Medical Sciences, Newcastle University, Newcastle upon Tyne, UK

5. NIHR Newcastle Biomedical Research Centre, Newcastle upon Tyne Hospitals NHS Foundation Trust, Cumbria, Northumberland, Tyne and Wear NHS Foundation Trust and Newcastle University, Newcastle upon Tyne, UK

6. Institute of Inflammation and Ageing, University of Birmingham, Birmingham, UK.

7. University College London Hospitals NHS Foundation Trust, London, UK and Bloomsbury Institute for Intensive Care Medicine, London, UK

8. Public Co-Investigator, ADMISSION Research Collaborative, Newcastle upon Tyne, UK

9. Population Health Sciences Institute, Faculty of Medical Sciences, Newcastle University, Newcastle upon Tyne, UK

10. Institute of Applied Health Research, University of Birmingham, Birmingham, UK

11. Population Health Sciences Institute, Faculty of Medical Sciences, Newcastle University, Newcastle upon Tyne, UK

12. School of Computing, Newcastle University, Newcastle upon Tyne, UK

13. Division of Population Health and Genomics, Ninewells Hospital and School of Medicine, University of Dundee, Dundee, UK

14. Biostatistics Research Group, Population Health Sciences Institute, Newcastle University, Newcastle upon Tyne, UK

ICD-10 codes used to identify conditions

a. List of comorbid conditions with ICD-10 codes used to identify each condition

Stroke	I61, I63.0, I63.1, I63.2, I63.3, I63.4, I63.5, I63.8, I63.9, I69.1, I69.3, G46.3, G46.4, G46.5, G46.6, G46.7, G46.8, I64, I65, I69.4
Transient Ischaemic Attack	G45.0, G45.1, G45.2, G45.3, G45.4, G45.8, G45.9, G46.0, G46.1, G46.2, I65, I66
Subarachnoid haemorrhage	I60, I69.0
Carpal tunnel syndrome	G56.0
Fibromyalgia	M79.7
Disc problem	M50, M51
Prolapsed disc/slipped disc	M50, M51
Ankylosing spondylitis	M45
Osteoarthritis	M15, M16, M17, M18, M19
Gout	M10, M14.0
Trigeminal neuralgia	G50.0
Disc degeneration	M50, M51
Hypertension	I10, I11, I12, I13, I15
Depression	F32, F33
Asthma	J45, J46
Atrial fibrillation	I48
Myocardial infarction	I252, I21, I22, I23, I24.1
Angina	I20.0 I20.1, I20.8, I20.9
Gastro-oesophageal reflux	K21
Oesophagitis	K20, K21.0, K22.1
Barretts oesophagus	K22.7
Gastric ulcer	K25
Gastritis	K29
Duodenal ulcer	K26
Diabetes mellitus	E10, E11, E12, E13, E14, G59.0, G63.2, H28.0, H35.0, H36.0, M14.2, N08.3, O24.0, O24.1, O24.2, O24.3
Hyperthyroidism/thyrotoxicosis	E05.0, E05.1, E05.2, E05.5, E05.8, E05.9, E06.2
Hypothyroidism/myxoedema	E03.5, E03.8, E03.9
Systemic Lupus Erythematosus	L93, M32
Sjogrens syndrome/sicca syndrome	M35.0
Scleroderma/systemic sclerosis	M34
Rheumatoid arthritis	J99.0, M05, M06
Psoriatic arthropathy	M07.0, M07.1, M07.2, M07.3, M09.0, L40.5
Polymyalgia Rheumatica	M31.5, M35.3
Malabsorption/coeliac disease	K90.0
COPD	J40, J41, J42, J43, J44
Anxiety/panic attacks	F40, F41
Obsessive compulsive disorder	F42
Irritable bowel syndrome	K58
Alcohol dependency	I42.6

Alcoholic liver disease/alcoholic cirrhosis	K70
Renal/kidney failure	N18.5
Kidney nephropathy	N00.0
Diverticular disease	K38.2, K57
Diverticulitis	K38.2, K57
Peripheral vascular disease	I73.1, I73.8, I73.9, I74.3, I74.4, I74.5
Leg claudication/intermittent claudication	I73.1, I73.8, I73.9, I74.3, I74.4, I74.5
Cardiomyopathy	I42.0, I42.6, I25.5, I42.3, I42.5, I42.7, I42.8, I42.9, I43
Hypertrophic cardiomyopathy	I42.1, I42.2
Heart failure/pulmonary oedema	I11.0. I13.0, I13.2, I50
Prostate problem (not cancer)	N40
Enlarged prostate	N40
Benign prostatic hypertrophy	N40
Glaucoma	H40.1, H40.2, H40.9
Epilepsy	G40, G41
Dementia	F00, F01, F03, F05.1, G30, F02.3
Schizophrenia	F20, F21, F22, F25, F28, F29
Mania/bipolar disorder	F30, F31
Manic depression	F30, F31
Dermatitis	L20, L23, L24, L25, L26, L27, L28, L30.0, L30.1, L30.2, L30.5, L30.8, L30.9,
Psoriasis	L40, M07.0, M07.2, M07.3, M09.0
Crohn’s disease	K50
Ulcerative colitis	K51
Migraine	G43
Chronic sinusitis	J32
Anorexia	F50.0, F50.1
Bulimia	F50.2, F50.3
Bronchiectasis	J47, Q33.4
Parkinson's disease	F02.3, G20
Multiple sclerosis	G35
Infective/viral hepatitis	B18
Hepatitis B	B18
Hepatitis C	B18
Hepatitis D	B18
Oesophageal varices	I85, I98.2, I98.3
Liver failure/cirrhosis	K70.1, K70.2, K70.3, K70.4, K71.1, K71.7, K72, K74.0, K74.1, K74.2, K74.4, K74.5, K74.6, K76.2, K76.3, B15.0, B16.0, B19.0
Osteoporosis	M80, M81, M82
Chronic fatigue syndrome	G93.3, F48.0
Endometriosis	N80
Meniere's disease	H81.0
Pernicious anaemia	D51
Polycystic ovary	E28.2

Lifetime diagnosis of cancer	Any C code, D00, D01, D02, D03, D04, D05, D06, D07, D08, D09, D49
Fragility fracture	S72.0, S72.1, S72.2, S52.5, S52.6, M48.5, M49.5
Incontinence	N39.3, N39.4
Delirium	F05

b. ICD-10 codes used to identify admissions for index conditions studied:

COPD exacerbation:

- J441,
- 195951007 (SNOMED),
- 196001008 (SNOMED)

Acute stroke:

- I63[subcodes 01234589],
- I693,
- I61
- I691,
- G46[subcodes 345678],
- I64
- I694
- G45[subcodes 0123489]
- G46[subcodes 012]
- I65
- I66
