# Supplementary material for: How far back do we need to look to capture diagnoses in electronic health records? A retrospective observational study of hospital electronic health record data
Source: BMJ Open. 2024 Feb 13;14(2):e080678. doi: 10.1136/bmjopen-2023-080678 (PMC10868273; doi:10.1136/bmjopen-2023-080678)
Supplement: Author's manuscript [file bmjopen-2023-080678.draft_revisions.pdf]

BMJ Open

BMJ Open is committed to open peer review. As part of this commitment we make the peer review history of every article we publish publicly available.

When an article is published we post the peer reviewers' comments and the authors' responses online. We also post the versions of the paper that were used during peer review. These are the versions that the peer review comments apply to.

The versions of the paper that follow are the versions that were submitted during the peer review process. They are not the versions of record or the final published versions. They should not be cited or distributed as the published version of this manuscript.

BMJ Open is an open access journal and the full, final, typeset and author-corrected version of record of the manuscript is available on our site with no access controls, subscription charges or pay-per-view fees (http://bmjopen.bmj.com).

If you have any questions on BMJ Open's open peer review process please email info.bmjopen@bmj.com

BMJ Open

How far back do we need to look to capture diagnoses in hospital electronic health records? An analysis of electronic hospital record data

Journal:	*BMJ Open*
Manuscript ID	bmjopen-2023-080678
Article Type:	Original research
Date Submitted by the Author:	07-Oct-2023

Complete List of Authors:	Lewis, Jadene; University of Birmingham, Health Informatics; University Hospitals Birmingham NHS Foundation Trust, Health Informatics<br>Evison, Felicity; University Hospitals Birmingham NHS Foundation Trust, Department of Health Informatics<br>Doal, Rominique; University of Birmingham, Health Informatics; University Hospitals Birmingham NHS Foundation Trust, Health Informatics<br>Field, Joanne; Newcastle Upon Tyne Hospitals NHS Foundation Trust<br>Gallier, Suzy; University Hospitals Birmingham NHS Foundation Trust, Health Informatics; University of Birmingham, PIONEER Technical Director<br>Harris, Steve; University College London Hospitals NHS Foundation Trust, Critical Care Department; University College London, Critical Care Department<br>le Roux, Peta; Newcastle Upon Tyne Hospitals NHS Foundation Trust<br>Osman, Mohammed; Newcastle University; Newcastle University<br>Plummer, Chris; Newcastle Upon Tyne Hospitals NHS Foundation Trust<br>Sapey, Elizabeth; University of Birmingham, Institute of Inflammation and Ageing<br>Singer, Mervyn; UCL, Bloomsbury Inst of Intensive Care Medicine<br>Sayer, Avan; Newcastle Upon Tyne Hospitals NHS Foundation Trust, NIHR Biomedical Research Centre; Newcastle University, Campus for Ageing and Vitality<br>Witham, Miles; Newcastle University; Newcastle University Research Collaborative, The ADMISSION; Newcastle University

Keywords:	Electronic Health Records, Information Extraction < Information Storage and Retrieval, Hospitals

SCHOLARONE™
Manuscripts

BMJ

I, the Submitting Author has the right to grant and does grant on behalf of all authors of the Work (as defined in the below author licence), an exclusive licence and/or a non-exclusive licence for contributions from authors who are: i) UK Crown employees; ii) where BMJ has agreed a CC-BY licence shall apply, and/or iii) in accordance with the terms applicable for US Federal Government officers or employees acting as part of their official duties; on a worldwide, perpetual, irrevocable, royalty-free basis to BMJ Publishing Group Ltd ("BMJ") its licensees and where the relevant Journal is co-owned by BMJ to the co-owners of the Journal, to publish the Work in this journal and any other BMJ products and to exploit all rights, as set out in our licence.

The Submitting Author accepts and understands that any supply made under these terms is made by BMJ to the Submitting Author unless you are acting as an employee on behalf of your employer or a postgraduate student of an affiliated institution which is paying any applicable article publishing charge ("APC") for Open Access articles. Where the Submitting Author wishes to make the Work available on an Open Access basis (and intends to pay the relevant APC), the terms of reuse of such Open Access shall be governed by a Creative Commons licence – details of these licences and which Creative Commons licence will apply to this Work are set out in our licence referred to above.

Other than as permitted in any relevant BMJ Author's Self Archiving Policies, I confirm this Work has not been accepted for publication elsewhere, is not being considered for publication elsewhere and does not duplicate material already published. I confirm all authors consent to publication of this Work and authorise the granting of this licence.

For peer review only

How far back do we need to look to capture diagnoses in hospital electronic health records? An analysis of electronic hospital record data

Short title: Lookback in hospital electronic health records

Jadene Lewis1

Felicity Evison1

Rominique Doal1

Joanne Field2

Suzy Gallier1

Steve Harris3

Peta le Roux2

Mohammed Osman4,5

Chris Plummer2,5

Elizabeth Sapey1,6

Mervyn Singer7

Avan Aihie Sayer4,5

Miles D Witham4,5

The ADMISSION Research Collaborative

1. PIONEER Hub, University of Birmingham, Birmingham, UK and Health Informatics, University Hospitals Birmingham NHS Foundation Trust, Birmingham, UK
2. Digital Services, Newcastle upon Tyne Hospitals NHS Foundation Trust, Newcastle upon Tyne, UK
3. Critical Care Department, University College London Hospitals NHS Foundation Trust, London, UK and Institute of Health Informatics, University College London, London, UK
4. AGE Research Group, Translational and Clinical Research Institute, Faculty of Medical Sciences, Newcastle University, Newcastle upon Tyne, UK
5. NIHR Newcastle Biomedical Research Centre, Newcastle upon Tyne Hospitals NHS Foundation Trust, Cumbria, Northumberland, Tyne and Wear NHS Foundation Trust and Newcastle University, Newcastle upon Tyne, UK
6. Institute of Inflammation and Ageing, University of Birmingham, Birmingham, UK.
7. University College London Hospitals NHS Foundation Trust, London, UK and Bloomsbury Institute for Intensive Care Medicine, London, UK

For peer review only - http://bmjopen.bmj.com/site/about/guidelines.xhtml

Correspondence to: Professor Miles Witham, AGE Research Group, NIHR Newcastle Biomedical Research Centre, 3rd floor, Biomedical Research Building, Campus for Ageing and Vitality, Newcastle upon Tyne, NE4 5PL. Tel: 0191 282 1317. Email: Miles.Witham@newcastle.ac.uk

Word count:

Abstract: 260 words

Main text: 1802

For peer review only

Abstract

Objectives:

To ascertain how far back in time electronic hospital records need to be interrogated to capture long-term condition diagnoses.

Design

Analysis of routinely collected hospital electronic health record data

Setting

University Hospitals Birmingham (UK) data held by the PIONEER acute care data hub.

Participants

We included data from the first recorded admission in 2018 with at least 10 years of lookback data in two datasets (COPD and acute stroke)

Outcome measures

We studied the most common ICD-10 coded diagnoses recorded in each dataset. For each diagnosis, we derived the number of patients with the diagnosis recorded at least once over the full 10-year lookback period, and then compared this with shorter lookback periods from 1 year to 9 years prior to the index admission.

Results

We included 560 patients with a full 10 years of lookback data in the COPD dataset, and 2142 patients in the stroke dataset. 7 of the top 10 commonest diagnoses in the COPD dataset reached >90% completeness by six years of lookback. Atrial fibrillation and diabetes were >90% coded with 2 to 3

For peer review only - http://bmjopen.bmj.com/site/about/guidelines.xhtml

For peer review only

years of lookback, but hypertension and asthma completeness continued to rise all the way out to 10 years of lookback. For stroke, 4 of the top 10 reached 90% completeness by 5 years of lookback; angina pectoris was >90% coded at 7 years and previous transient ischaemic attack completeness continued to rise out to 10 years of lookback.

Conclusion

A seven-year lookback captures most, but not all common diagnoses. The duration of lookback should be tailored to the conditions being studied.

Strengths and limitations of this study

- We analysed common conditions associated with two different index conditions underlying hospital admission
- Our analysis was embedded within a mature, research active electronic health record, enhancing the applicability of the findings for future research
- Less common associated conditions were not studied, and may require different lookback periods for effective capture
- Our analysis was confined to a single electronic hospital record and the required lookback periods could differ between hospitals

Keywords

Electronic health record; multimorbidity; multiple long-term conditions; diagnostic coding

For peer review only

Background

Multiple long-term conditions (MLTC) (multimorbidity) are an important but understudied challenge to healthcare systems.[1] Much of the research on MLTC to date has used large population-based datasets or has focussed on primary care data; there has been comparatively little work on MLTC within the hospital environment.[2] Understanding the associations, consequences, and pathways of care for MLTC within hospital is critical if we are to understand how best to prevent the adverse consequences of MLTC and deliver better care for those living with MLTC admitted to hospital.[3]

Analysis of routinely-collected electronic healthcare records affords the opportunity to study MLTC at scale, including in groups of patients (e.g. those with dementia and those with critical illness) who would not be able to join traditional consented studies. Central to the study of MLTC in patients admitted to hospital is the accurate identification of which long-term conditions affect hospitalised patients. In the UK, diagnoses within EHR are recorded at discharge but can also be recorded during admissions. The quality and completeness of diagnostic coding is highly variable however,[4,5] and examining diagnoses coded during a single admission may not provide a complete picture of all diagnoses affecting an individual.

Aggregating diagnoses from a series of hospital admissions provides an opportunity to improve the completeness of recording, but it is unclear how far back through an electronic healthcare record researchers should look. Different EHRs have been in existence for different amounts of time, and comparison between EHRs may introduce bias if different lookback times introduce differences in the completeness of diagnostic recording.

The aim of this analysis was therefore to investigate how far back we need to look in order to capture the majority of diagnoses documented from previous admissions, and whether there are differences between diagnoses in what the minimum lookback period should be.

For peer review only - http://bmjopen.bmj.com/site/about/guidelines.xhtml

Methods

Hospital and data sources

The Queen Elizabeth Hospital Birmingham (QEHB) is a National Health Service acute hospital providing secondary and tertiary care services to adults in a large urban and suburban catchment in England, UK. The hospital has 1269 beds including 80 level 2/3 intensive care beds and an Emergency Department that assesses >300 patients per day. QEHB uses an electronic healthcare record (EHR) (PICS, Birmingham Systems) containing time-stamped, structured records including demography, location, time of admission and discharge, all treatments and investigations, and physiological measurements derived from the NEWS2 national early warning score. Diagnoses are coded using SNOMED-CT. The EHR has been in place since 1999 and the Trust is a paperless environment for all care provision and planning.

For this analysis, we used two data extracts. We extracted data on all acute admissions between 1st January 2018 and 31st December 2018 with a discharge diagnosis of COPD exacerbation (as a primary or secondary diagnosis). Using this time period enabled us to avoid any distorting effects from the COVID-19 pandemic. We took the date of the first hospital admission for COPD exacerbation in 2018 as the index date, and extracted data on age, sex and all hospital diagnoses coded by ICD-10 discharge codes within ten years prior to the admission under study. For the second data extract, we repeated the procedure but extracted data on all acute admissions with a discharge diagnosis of stroke.

Approval by PIONEER Data Trust Committee

This study was approved under umbrella ethics and Confidentiality Advisory Group (CAG) approvals which underpin the work of PIONEER, the Health Data Research (HDR)-UK Hub in Acute Care (East Midlands–Derby Research Ethics Committee reference: 20/EM/0158 and CAG reference: 20/CAG/0084). This Section 251 support via the NHS Health Research Authority Confidentiality

For peer review only - http://bmjopen.bmj.com/site/about/guidelines.xhtml

Advisory Group gives permission for the use of routinely collected clinical data for research without Individual participant consent. As part of this process, the data access request and specification were reviewed and approved by the PIONEER Data Trust Committee, comprising a group of patients and members of the public who provide oversight of all data access decisions within PIONEER.[6]

Patient and public involvement

The ADMISSION grant programme was co-designed with a panel of patients including patients with MLTC. Two public co-applicants form part of the management team for the ADMISSION programme, and studies within ADMISSION including this analysis are discussed with the ADMISSION Patient Advisory Group, who provide input on the design, interpretation and dissemination of results.

Analyses

We derived diagnoses made during, or at discharge, from ICD-10 codes recorded in the EHR over the 10 years prior to the index admission date. Code lists used for each diagnosis are given in Supplementary Material; the conditions selected were based on conditions previously used in UK BioBank,[7] modified to remove diagnoses less likely to be coded in hospital discharge data. We then examined the percentage of diagnoses that were captured by looking back from the date of index admission for increasing periods of time from 1 year to 10 years and compared the proportion of patients with diagnoses captured for each lookback period with the number of diagnoses captured with 10 years of lookback. Only individuals with a full 10 years of EHR data were included in the analyses.

Results

We included 560 individuals in the COPD exacerbation analysis dataset (32 were excluded due to incomplete lookback data), and 2142 individuals in the acute stroke dataset (1899 were excluded due to incomplete lookback data). A total of 73 different diagnoses were recorded for those with COPD

For peer review only - http://bmjopen.bmj.com/site/about/guidelines.xhtml

exacerbation (median 3 per individual) and a total of 80 different diagnoses were recorded for those with acute stroke (median 6 per individual). Table 1 shows details of individuals in each dataset, along with the top 10 most common diagnoses recorded in each dataset.

Figure 1 shows the percentage of each condition detected per year of lookback in the COPD dataset, using the number recorded over the full 10 year lookback period as reference. Considering all conditions together, 90% of conditions were recorded by 6 years of lookback. Seven of the top 10 reached 90% completeness by 6 years but two conditions (asthma and hypertension) continued to show increases in coding completeness with longer lookback all the way out to 10 years. Figure 2 shows similar data for the acute stroke dataset; overall 90% of diagnoses had been recorded by 5 years of lookback, but there was considerable heterogeneity, from dementia (90% by 3 years) and myocardial infarction (90% by 4 years) through to diabetes mellitus (90% by 5 years) and angina pectoris (90% by 7 years). Previous transient ischaemic attack coding completeness continued to rise out to 10 years with no flattening of the curve.

Discussion

Our results suggest that a seven year lookback is sufficient to capture some but not all common diagnoses for patients admitted with COPD exacerbations or acute stroke. For most diagnoses investigated, the prevalence curves flatten out between six and seven years, suggesting that additional years of lookback adds few additional diagnoses. There were however notable exceptions, particularly hypertension and asthma in the COPD cohort, and previous TIA in the acute stroke cohort. For these diagnoses, curves did not flatten at ten years, suggesting that even longer periods of lookback may be required to gain complete diagnostic data. These data suggest that the duration of lookback needs to be tailored to the condition of interest.

For peer review only

Our results give a useful indication of what a minimum lookback time in hospital electronic health records might need to be, but they also highlight that for some diagnoses, reliance on coded, structured diagnostic data is likely to remain inadequate. Not all diagnoses are written down in hospital records, and for those that are, many are not recorded or coded in a structured manner.[8] It is these diagnoses that require additional work to extract, either from unstructured EHR data using techniques such as natural language processing,[9] by using additional data sources, for instance prescribing records[10] or laboratory data,[11] and by linkage to alternative sources of diagnostic data (for instance data from primary care).[12] Our results help to highlight diagnoses for which this work is particularly needed.

Strengths of our approach include the use of routinely collected electronic healthcare data and the interrogation of two common diagnostic reasons for hospital admission. We were limited by an inability to go back further than 10 years without a reduction in the number of patients included in the analysis, and we have confined our analysis to the most common conditions associated with each index condition – other, less common conditions may require different lookback periods. Finally, our analysis is confined to a single electronic health record, and lookback times may vary across different healthcare organisations – organisations may vary in how well they code different diagnoses. These issues provide a starting point for future research in this area.

In summary, whilst a seven year lookback appears adequate to capture some common diagnoses associated with COPD exacerbation and acute stroke admissions, there is considerable variability between diagnoses. Researchers need to test the completeness of specific diagnoses in specific EHR systems that they use to be sure that diagnosis capture is suitably complete for the conditions they wish to study.

Funding

For peer review only - http://bmjopen.bmj.com/site/about/guidelines.xhtml

This research was conducted as part of the ADMISSION research collaborative, funded by the Strategic Priority Fund "Tackling multimorbidity at scale" programme [grant number MR/V033654/1]. This funding is delivered by the Medical Research Council and the National Institute for Health and Care Research in partnership with the Economic and Social Research Council and in collaboration with the Engineering and Physical Sciences Research Council. The views expressed in this publication are those of the authors and not necessarily those of UK Research and Innovation, the National Institute for Health and Care Research, or the Department of Health and Social Care.

Competing Interests

ES is director of the PIONEER acute data hub, funded by Health Data Research-UK. The other authors have no conflicts of interest to declare.

Author statement

Conception and design: JL, MDW, CP, ES, MS, AAS

Data curation and analysis: JL, FE, RD, SG, JF, SH, PLR, MO

Interpretation of results: MDW, AAS, FE, AAS

Manuscript draft: JL, MDW

Critical revision of manuscript: All authors

Data statement

The data that support the findings of this study are not openly available due to reasons of sensitivity. Data may be accessed on request to the HDRUK PIONEER Acute Data Hub on provision of permission from the PIONEER Data Trust Committee and provision of a data access agreement. Data are located in controlled access data storage at the PIONEER acute data hub.

Acknowledgements

For peer review only

This work used data provided by patients and collected by the NHS as part of their care and support. MDW, CP and AAS acknowledge support from the National Institute for Health and Care Research (NIHR) Newcastle Biomedical Research Centre based at Newcastle upon Tyne Hospitals NHS Foundation Trust, Cumbria, Northumberland, Tyne and Wear NHS Foundation Trust and Newcastle University.

References

[1] Whitty CJ. Harveian Oration 2017: Triumphs and challenges in a world shaped by medicine. Clin Med (Lond) 2017;17:537-544.

[2] Kudesia P, Salimarouny B, Stanley M, et al. The incidence of multimorbidity and patterns in accumulation of chronic conditions: A systematic review. J Comorb 2021;11:26335565211032880.

[3] Owen N, Dew L, Logan S, Denegri S, Chappell LC. Research policy for people with multiple long-term conditions and their carers. J Multimorb Comorb 2022;12:26335565221104407.

[4] Ugboma I, Syddall HE, Cox V, Cooper C, Briggs R, Sayer AA. Coding Geriatric syndromes: How good are we? CME J Geriatr Med 2008;10:34-6.

[5] Wright A, McCoy AB, Hickman TT, et al. Problem list completeness in electronic health records: A multi-site study and assessment of success factors. Int J Med Inform 2015;84:784-90.

[6] Gallier S, Price G, Pandya H, et al. Infrastructure and operating processes of PIONEER, the HDR-UK Data Hub in Acute Care and the workings of the Data Trust Committee: a protocol paper. BMJ Health Care Inform 2021;28:e100294.

[7] Gallacher KI, McQueenie R, Nicholl B, Jani BD, Lee D, Mair FS. Risk factors and mortality associated with multimorbidity in people with stroke or transient ischaemic attack: a study of 8,751 UK Biobank participants. J Comorb 2018;8:1– 8.

[8] Todd OM, Burton JK, Dodds RM, et al. New Horizons in the use of routine data for ageing research. Age Ageing 2020;49:716-22.

For peer review only

For peer review only - http://bmjopen.bmj.com/site/about/guidelines.xhtml

[9] Jackson RG, Patel R, Jayatilleke N, et al. Natural language processing to extract symptoms of severe mental illness from clinical text: the Clinical Record Interactive Search Comprehensive Data Extraction (CRIS-CODE) project. BMJ Open 2017;7:e012012.

[10] Hansen ER, Sagi T, Hose K, Lip GYH, Larsen TB, Skjøth F. Assigning diagnosis codes using medication history. Artif Intell Med 2022;128:102307.

[11] Schulz WL, Young P, Coppi A, et al. Temporal relationship of computed and structured diagnoses in electronic health record data. BMC Med Inform Decis Mak 2021;21:61.

[12] Herrett E, Shah AD, Boggon R, et al. Completeness and diagnostic validity of recording acute myocardial infarction events in primary care, hospital care, disease registry, and national mortality records: cohort study. BMJ 2013;346:f2350.

For peer review only

For peer review only - http://bmjopen.bmj.com/site/about/guidelines.xhtml

Table 1: Study Population details

	COPD dataset		Stroke dataset	
N	560		2142	
Mean age (years) (SD)	71 (11)		73 (13)	
Female sex (%)	296 (52)		1064 (50)	
Median number of comorbid conditions (IQR)*	3 (1 to 5)		6 (4 to 8)	
Top 10 previously diagnosed conditions	Name	Frequency	Name	Frequency
1	COPD	560	Stroke	1729
2	Heart failure	227	Atrial fibrillation	843
3	Asthma	206	TIA	832
4	Atrial fibrillation	197	All Diabetes	804
5	Depression	182	Heart failure	631
6	Anxiety	180	COPD	525
7	All Diabetes	159	Myocardial infarction	519
8	Hypertension	151	Angina	491
9	Myocardial infarction	137	Dementia	432
10	Angina	126	Depression	426

*in addition to the index condition (COPD or stroke)

For peer review only

For peer review only - http://bmjopen.bmj.com/site/about/guidelines.xhtml

Figure legends

Figure 1. COPD exacerbation top 10 previously diagnosed conditions

Figure 2. Acute stroke top 10 previously diagnosed conditions

For peer review only

For peer review only - http://bmjopen.bmj.com/site/about/guidelines.xhtml

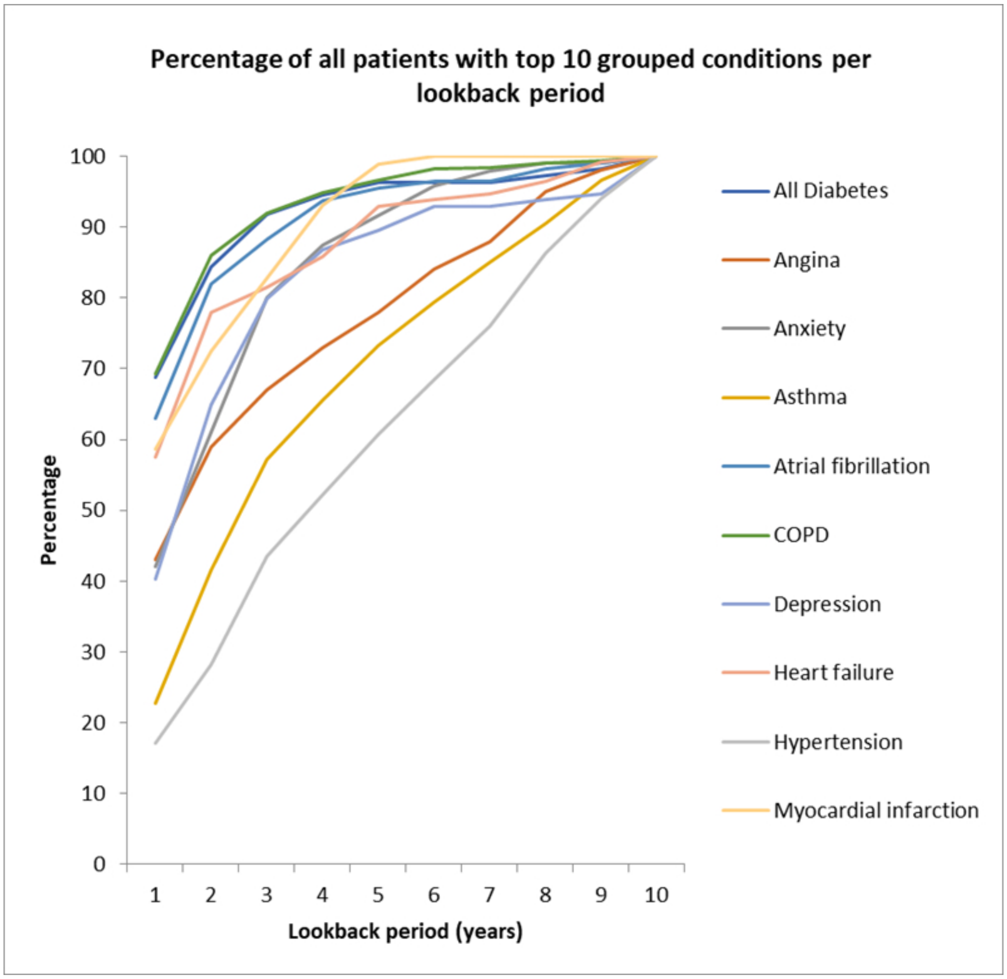

COPD exacerbation top 10 previously diagnosed conditions

343x333mm (59 x 59 DPI)

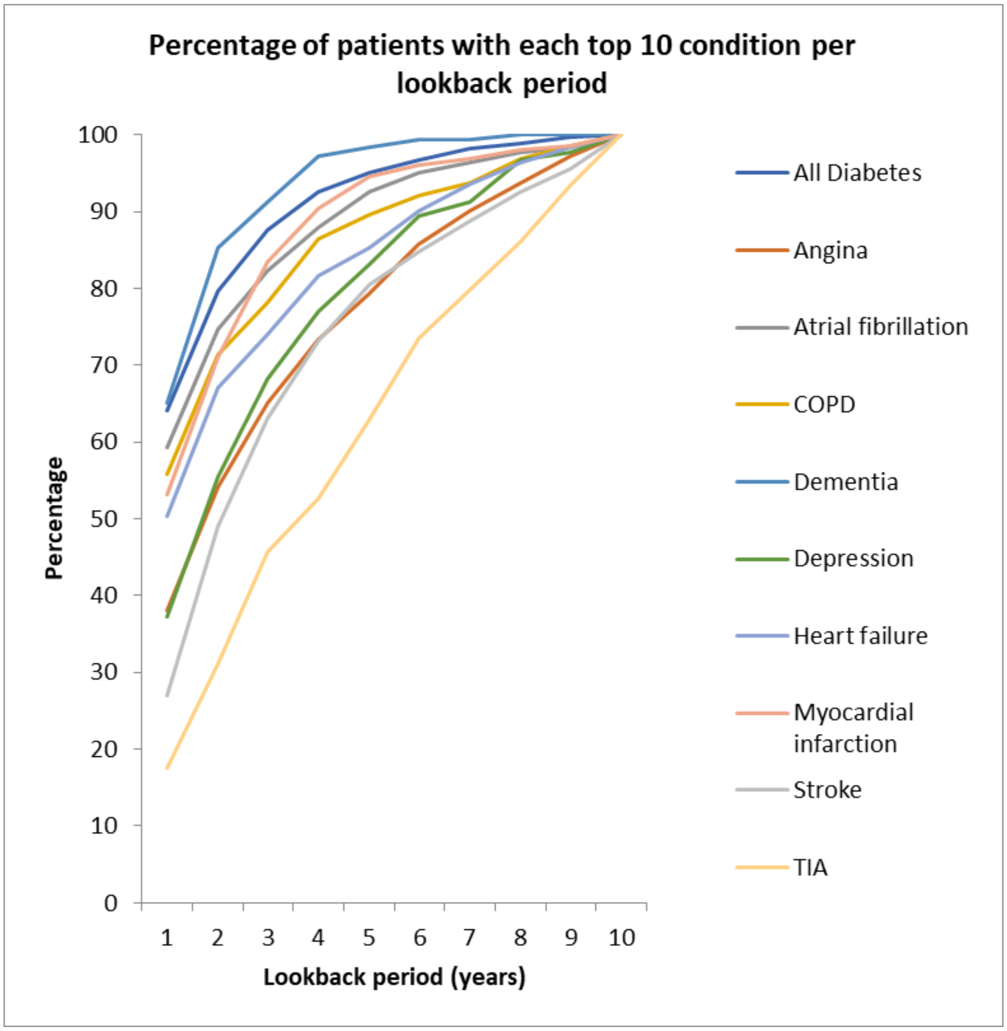

Acute stroke top 10 previously diagnosed conditions

338x346mm (59 x 59 DPI)

Supplementary Information

ADMISSION Research Collaborative Consortium members:

Avan Aihie Sayer[4,5]

Victoria Bartle[8]

Rachel Cooper[4,5]

Heather J Cordell[9]

Ray Holding[8]

Tom Marshall[10]

Fiona E Matthews[11]

Paolo Missier[12]

Ewan Pearson[13]

Chris Plummer[2,5]

Sian Robinson[4,5]

Elizabeth Sapey[1,6]

Mervyn Singer[7]

Thomas Scharf[11]

James Wason[14]

Miles D Witham[4,5]

1. PIONEER Hub, University of Birmingham, Birmingham, UK and Health Informatics, University Hospitals Birmingham NHS Foundation Trust, Birmingham, UK

2. Digital Services, Newcastle upon Tyne Hospitals NHS Foundation Trust, Newcastle upon Tyne, UK

4. AGE Research Group, Translational and Clinical Research Institute, Faculty of Medical Sciences, Newcastle University, Newcastle upon Tyne, UK

For peer review only

5. NIHR Newcastle Biomedical Research Centre, Newcastle upon Tyne Hospitals NHS Foundation Trust, Cumbria, Northumberland, Tyne and Wear NHS Foundation Trust and Newcastle University, Newcastle upon Tyne, UK

6. Institute of Inflammation and Ageing, University of Birmingham, Birmingham, UK.

7. University College London Hospitals NHS Foundation Trust, London, UK and Bloomsbury Institute for Intensive Care Medicine, London, UK

8. Public Co-Investigator, ADMISSION Research Collaborative, Newcastle upon Tyne, UK

9. Population Health Sciences Institute, Faculty of Medical Sciences, Newcastle University, Newcastle upon Tyne, UK

10. Institute of Applied Health Research, University of Birmingham, Birmingham, UK

11. Population Health Sciences Institute, Faculty of Medical Sciences, Newcastle University, Newcastle upon Tyne, UK

12. School of Computing, Newcastle University, Newcastle upon Tyne, UK

13. Division of Population Health and Genomics, Ninewells Hospital and School of Medicine, University of Dundee, Dundee, UK

14. Biostatistics Research Group, Population Health Sciences Institute, Newcastle University, Newcastle upon Tyne, UK

For peer review only

ICD-10 codes used to identify conditions

a. List of comorbid conditions with ICD-10 codes used to identify each condition

Stroke	I61, I63.0, I63.1, I63.2, I63.3, I63.4, I63.5, I63.8, I63.9, I69.1, I69.3, G46.3, G46.4, G46.5, G46.6, G46.7, G46.8, I64, I65, I69.4
Transient Ischaemic Attack	G45.0, G45.1, G45.2, G45.3, G45.4, G45.8, G45.9, G46.0, G46.1, G46.2, I65, I66
Subarachnoid haemorrhage	I60, I69.0
Carpal tunnel syndrome	G56.0
Fibromyalgia	M79.7
Disc problem	M50, M51
Prolapsed disc/slipped disc	M50, M51
Ankylosing spondylitis	M45
Osteoarthritis	M15, M16, M17, M18, M19
Gout	M10, M14.0
Trigeminal neuralgia	G50.0
Disc degeneration	M50, M51
Hypertension	I10, I11, I12, I13, I15
Depression	F32, F33
Asthma	J45, J46
Atrial fibrillation	I48
Myocardial infarction	I252, I21, I22, I23, I24.1
Angina	I20.0 I20.1, I20.8, I20.9
Gastro-oesophageal reflux	K21
Oesophagitis	K20, K21.0, K22.1
Barretts oesophagus	K22.7
Gastric ulcer	K25
Gastritis	K29
Duodenal ulcer	K26
Diabetes mellitus	E10, E11, E12, E13, E14, G59.0, G63.2, H28.0, H35.0, H36.0, M14.2, N08.3, O24.0, O24.1, O24.2, O24.3
Hyperthyroidism/thyrotoxicosis	E05.0, E05.1, E05.2, E05.5, E05.8, E05.9, E06.2
Hypothyroidism/myxoedema	E03.5, E03.8, E03.9
Systemic Lupus Erythematosus	L93, M32
Sjogrens syndrome/sicca syndrome	M35.0
Scleroderma/systemic sclerosis	M34
Rheumatoid arthritis	J99.0, M05, M06
Psoriatic arthropathy	M07.0, M07.1, M07.2, M07.3, M09.0, L40.5
Polymyalgia Rheumatica	M31.5, M35.3
Malabsorption/coeliac disease	K90.0
COPD	J40, J41, J42, J43, J44
Anxiety/panic attacks	F40, F41
Obsessive compulsive disorder	F42
Irritable bowel syndrome	K58
Alcohol dependency	I42.6

For peer review only

Alcoholic liver disease/alcoholic cirrhosis	K70
Renal/kidney failure	N18.5
Kidney nephropathy	N00.0
Diverticular disease	K38.2, K57
Diverticulitis	K38.2, K57
Peripheral vascular disease	I73.1, I73.8, I73.9, I74.3, I74.4, I74.5
Leg claudication/intermittent claudication	I73.1, I73.8, I73.9, I74.3, I74.4, I74.5
Cardiomyopathy	I42.0, I42.6, I25.5, I42.3, I42.5, I42.7, I42.8, I42.9, I43
Hypertrophic cardiomyopathy	I42.1, I42.2
Heart failure/pulmonary oedema	I11.0. I13.0, I13.2, I50
Prostate problem (not cancer)	N40
Enlarged prostate	N40
Benign prostatic hypertrophy	N40
Glaucoma	H40.1, H40.2, H40.9
Epilepsy	G40, G41
Dementia	F00, F01, F03, F05.1, G30, F02.3
Schizophrenia	F20, F21, F22, F25, F28, F29
Mania/bipolar disorder	F30, F31
Manic depression	F30, F31
Dermatitis	L20, L23, L24, L25, L26, L27, L28, L30.0, L30.1, L30.2, L30.5, L30.8, L30.9,
Psoriasis	L40, M07.0, M07.2, M07.3, M09.0
Crohn’s disease	K50
Ulcerative colitis	K51
Migraine	G43
Chronic sinusitis	J32
Anorexia	F50.0, F50.1
Bulimia	F50.2, F50.3
Bronchiectasis	J47, Q33.4
Parkinson's disease	F02.3, G20
Multiple sclerosis	G35
Infective/viral hepatitis	B18
Hepatitis B	B18
Hepatitis C	B18
Hepatitis D	B18
Oesophageal varices	I85, I98.2, I98.3
Liver failure/cirrhosis	K70.1, K70.2, K70.3, K70.4, K71.1, K71.7, K72, K74.0, K74.1, K74.2, K74.4, K74.5, K74.6, K76.2, K76.3, B15.0, B16.0, B19.0
Osteoporosis	M80, M81, M82
Chronic fatigue syndrome	G93.3, F48.0
Endometriosis	N80
Meniere's disease	H81.0
Pernicious anaemia	D51
Polycystic ovary	E28.2

For peer review only

Lifetime diagnosis of cancer	Any C code, D00, D01, D02, D03, D04, D05, D06, D07, D08, D09, D49
Fragility fracture	S72.0, S72.1, S72.2, S52.5, S52.6, M48.5, M49.5
Incontinence	N39.3, N39.4
Delirium	F05

b. ICD-10 codes used to identify admissions for index conditions studied:

COPD exacerbation:

- J441,
- 195951007 (SNOMED),
- 196001008 (SNOMED)

Acute stroke:

- I63[subcodes 01234589],
- I693,
- I61
- I691,
- G46[subcodes 345678],
- I64
- U543
- G45[subcodes 0123489]
- G46[subcodes 012]
- I65
- I66

For peer review only

BMJ Open

How far back do we need to look to capture diagnoses in electronic health records? An retrospective observational study of hospital electronic health record data

Journal:	*BMJ Open*
Manuscript ID	bmjopen-2023-080678.R1
Article Type:	Original research
Date Submitted by the Author:	28-Dec-2023
Complete List of Authors:	Lewis, Jadene; University of Birmingham, Health Informatics; University Hospitals Birmingham NHS Foundation Trust, Health Informatics<br>Evison, Felicity; University Hospitals Birmingham NHS Foundation Trust, Department of Health Informatics<br>Doal, Rominique; University of Birmingham, Health Informatics; University Hospitals Birmingham NHS Foundation Trust, Health Informatics<br>Field, Joanne; Newcastle Upon Tyne Hospitals NHS Foundation Trust<br>Gallier, Suzy; University Hospitals Birmingham NHS Foundation Trust, Health Informatics; University of Birmingham, PIONEER Technical Director<br>Harris, Steve; University College London Hospitals NHS Foundation Trust, Critical Care Department; University College London, Critical Care Department<br>le Roux, Peta; Newcastle Upon Tyne Hospitals NHS Foundation Trust<br>Osman, Mohammed; Newcastle University; Newcastle University<br>Plummer, Chris; Newcastle Upon Tyne Hospitals NHS Foundation Trust<br>Sapey, Elizabeth; University of Birmingham, Institute of Inflammation and Ageing<br>Singer, Mervyn; UCL, Bloomsbury Inst of Intensive Care Medicine<br>Sayer, Avan; Newcastle Upon Tyne Hospitals NHS Foundation Trust, NIHR Biomedical Research Centre; Newcastle University, Campus for Ageing and Vitality<br>Witham, Miles; Newcastle University; Newcastle University Research Collaborative, The ADMISSION; Newcastle University
**Primary Subject Heading**:	Research methods
Secondary Subject Heading:	Health informatics
Keywords:	Electronic Health Records, Information Extraction < Information Storage and Retrieval, Hospitals

SCHOLARONE™
Manuscripts

I, the Submitting Author has the right to grant and does grant on behalf of all authors of the Work (as defined in the below author licence), an exclusive licence and/or a non-exclusive licence for contributions from authors who are: i) UK Crown employees; ii) where BMJ has agreed a CC-BY licence shall apply, and/or iii) in accordance with the terms applicable for US Federal Government officers or employees acting as part of their official duties; on a worldwide, perpetual, irrevocable, royalty-free basis to BMJ Publishing Group Ltd ("BMJ") its licensees and where the relevant Journal is co-owned by BMJ to the co-owners of the Journal, to publish the Work in this journal and any other BMJ products and to exploit all rights, as set out in our licence.

The Submitting Author accepts and understands that any supply made under these terms is made by BMJ to the Submitting Author unless you are acting as an employee on behalf of your employer or a postgraduate student of an affiliated institution which is paying any applicable article publishing charge ("APC") for Open Access articles. Where the Submitting Author wishes to make the Work available on an Open Access basis (and intends to pay the relevant APC), the terms of reuse of such Open Access shall be governed by a Creative Commons licence – details of these licences and which Creative Commons licence will apply to this Work are set out in our licence referred to above.

Other than as permitted in any relevant BMJ Author's Self Archiving Policies, I confirm this Work has not been accepted for publication elsewhere, is not being considered for publication elsewhere and does not duplicate material already published. I confirm all authors consent to publication of this Work and authorise the granting of this licence.

For peer review only

How far back do we need to look to capture diagnoses in electronic health records? An retrospective observational study of hospital electronic health record data

Short title: Lookback in Queen Elizabeth Hospital Birmingham electronic health records

Jadene Lewis1

Felicity Evison1

Rominique Doal1

Joanne Field2

Suzy Gallier1

Steve Harris3

Peta le Roux2

Mohammed Osman4,5

Chris Plummer2,5

Elizabeth Sapey1,6

Mervyn Singer7

Avan Aihie Sayer4,5

Miles D Witham4,5

The ADMISSION Research Collaborative

1. PIONEER Hub, University of Birmingham, Birmingham, UK and Health Informatics, University Hospitals Birmingham NHS Foundation Trust, Birmingham, UK
2. Digital Services, Newcastle upon Tyne Hospitals NHS Foundation Trust, Newcastle upon Tyne, UK
3. Critical Care Department, University College London Hospitals NHS Foundation Trust, London, UK and Institute of Health Informatics, University College London, London, UK
4. AGE Research Group, Translational and Clinical Research Institute, Faculty of Medical Sciences, Newcastle University, Newcastle upon Tyne, UK
5. NIHR Newcastle Biomedical Research Centre, Newcastle upon Tyne Hospitals NHS Foundation Trust, Cumbria, Northumberland, Tyne and Wear NHS Foundation Trust and Newcastle University, Newcastle upon Tyne, UK
6. Institute of Inflammation and Ageing, University of Birmingham, Birmingham, UK.
7. University College London Hospitals NHS Foundation Trust, London, UK and Bloomsbury Institute for Intensive Care Medicine, London, UK

For peer review only - http://bmjopen.bmj.com/site/about/guidelines.xhtml

For peer review only

Correspondence to: Professor Miles Witham, AGE Research Group, NIHR Newcastle Biomedical Research Centre, 3rd floor, Biomedical Research Building, Campus for Ageing and Vitality, Newcastle upon Tyne, NE4 5PL. Tel: 0191 282 1317. Email: Miles.Witham@newcastle.ac.uk

Word count:

Abstract: 299 words

Main text: 1802

For peer review only

For peer review only - http://bmjopen.bmj.com/site/about/guidelines.xhtml

Abstract

Objectives:

Analysis of routinely-collected electronic health data is a key tool for long-term conditions research and practice for hospitalised patients. This requires accurate and complete ascertainment of a broad range of diagnoses; something not always recorded on an admission document at a single point in time. This study aimed to ascertain how far back in time electronic hospital records need to be interrogated to capture long-term condition diagnoses.

Design

Retrospective observational study of routinely collected hospital electronic health record data.

Setting

Queen Elizabeth Hospital Birmingham (UK) linked data held by the PIONEER acute care data hub.

Participants

Patients whose first recorded admission for COPD exacerbation (n=560) or acute stroke (n=2142) was between January and December 2018 and who had a minimum of 10 years of data prior to the index date.

Outcome measures

We identified the most common ICD-10 coded diagnoses received by COPD and acute stroke patients separately. For each diagnosis, we derived the number of patients with the diagnosis recorded at least once over the full 10-year lookback period, and then compared this with shorter lookback periods from 1 year to 9 years prior to the index admission.

For peer review only

Results

Seven of the top 10 commonest diagnoses in the COPD dataset reached >90% completeness by six years of lookback. Atrial fibrillation and diabetes were >90% coded with 2 to 3 years of lookback, but hypertension and asthma completeness continued to rise all the way out to 10 years of lookback. For stroke, 4 of the top 10 reached 90% completeness by 5 years of lookback; angina pectoris was >90% coded at 7 years and previous transient ischaemic attack completeness continued to rise out to 10 years of lookback.

Conclusion

A seven-year lookback captures most, but not all common diagnoses. Lookback duration should be tailored to the conditions being studied.

Strengths and limitations of this study

- We analysed common conditions associated with two different index conditions underlying hospital admission
- Our mature, large hospital dataset enabled us to study a variety of conditions and analyse longitudinal trends over ten years
- Less common associated conditions were not studied, and may require different lookback periods for effective capture
- Our analysis was confined to a single electronic hospital record and the required lookback periods could differ between hospitals

Keywords

Electronic health record; multimorbidity; multiple long-term conditions; diagnostic coding

For peer review only - http://bmjopen.bmj.com/site/about/guidelines.xhtml

Background

Multiple long-term conditions (MLTC) (multimorbidity) are an important but understudied challenge to healthcare systems. [1] MLTC, defined as two or more long-term health conditions, are common with an estimated global prevalence of 37%. [2] The prevalence in old age is even higher, reaching 80% by the age of 80 years in the UK. [3] Much of the research on MLTC to date has used large population-based datasets or has focussed on primary care data; there has been comparatively little work on MLTC within the hospital environment. [4] Understanding the associations, consequences, and pathways of care for MLTC within hospital is critical if we are to understand how best to prevent the adverse consequences of MLTC and deliver better care for those living with MLTC when they are admitted to hospital. [5]

Analysis of routinely-collected electronic healthcare records affords the opportunity to study MLTC at scale, across a wide range of patient groups, including those who would not be able to join traditional consented studies, such as those with dementia or with critical illness. Central to the study of MLTC in patients admitted to hospital is the accurate identification of which long-term conditions affect hospitalised patients. In the UK, diagnoses within Electronic Healthcare Records (EHRs) are recorded at discharge, but can also be recorded during admissions. The quality and completeness of diagnostic coding is highly variable however, [6,7] and examining diagnoses coded during a single admission may not provide a complete picture of all diagnoses affecting an individual.

Aggregating diagnoses from a series of hospital admissions provides an opportunity to improve the completeness of recording, but it is unclear how far back through an electronic healthcare record researchers should look. Different EHRs have been in existence for different amounts of time, and comparison between EHRs may introduce bias if different lookback times introduce differences in the completeness of diagnostic recording. Understanding the completeness of diagnostic recording for a given look back period would help to avoid under-recording of diagnoses in both research analyses

For peer review only - http://bmjopen.bmj.com/site/about/guidelines.xhtml

and clinical applications and would thus enable analysis teams to make informed judgements about what records and which conditions to study when analysing routinely-collected electronic health data.

The aim of this analysis was therefore to investigate how far back we need to look in order to capture the majority of diagnoses documented from previous admissions, and whether there are differences between diagnoses in what the minimum lookback period should be.

Methods

Hospital and data sources

The Queen Elizabeth Hospital Birmingham (QEHB) is a National Health Service acute hospital providing secondary and tertiary care services to adults in a large urban and suburban catchment area in England, UK. The hospital has 1269 beds including 80 level 2/3 intensive care beds and an Emergency Department that assesses >300 patients per day. QEHB uses an EHR (PICS, Birmingham Systems) containing time-stamped, structured records including demographics, location, time of admission and discharge, all treatments and investigations, and physiological measurements such as pulse, blood pressure and respiratory rate derived from the National Early Warning Score version 2 (NEWS2). Diagnoses are currently coded using the Systematised Nomenclature of Medicine – Clinical Terms (SNOMED-CT) system, and historically used International Classification of Diseases version 10 (ICD-10). The EHR has been in place since 1999 and the Trust is a paperless environment for all care provision and planning.

For this analysis, we used two data extracts. We extracted data on all acute admissions between 1st January 2018 and 31st December 2018 with a discharge diagnosis of COPD exacerbation (as a primary or secondary diagnosis). We also used SNOMED codes recorded in the electronic health record to resolve whether a diagnosis coded as 'COPD unspecified' was due to an acute exacerbation. Using this time period enabled us to avoid any distorting effects from the COVID-19 pandemic. We took the date

For peer review only - http://bmjopen.bmj.com/site/about/guidelines.xhtml

of the first hospital admission for COPD exacerbation in 2018 as the index date, and extracted data on age, sex and all hospital diagnoses coded by ICD-10 discharge codes within ten years prior to the admission under study. For the second data extract, we repeated the procedure but extracted data on all acute admissions with a discharge diagnosis of stroke. We chose COPD exacerbation and stroke as exemplar conditions as they are common causes of admission to hospital and are associated with a substantial burden of comorbidity.

Approval by PIONEER Data Trust Committee

This study was approved under umbrella ethics and Confidentiality Advisory Group (CAG) approvals which underpin the work of PIONEER, the Health Data Research (HDR)-UK Hub in Acute Care (East Midlands–Derby Research Ethics Committee reference: 20/EM/0158 and CAG reference: 20/CAG/0084). This Section 251 support via the NHS Health Research Authority Confidentiality Advisory Group gives permission for the use of routinely collected clinical data for research without individual participant consent. As part of this process, the data access request and specification were reviewed and approved by the PIONEER Data Trust Committee, comprising a group of patients and members of the public who provide oversight of all data access decisions within PIONEER. Governance and linkage processes for PIONEER data have been published previously. [8]

Patient and public involvement

The ADMISSION grant programme was co-designed with a panel of patients including patients with MLTC. Two public co-applicants form part of the management team for the ADMISSION programme, and studies within ADMISSION including this analysis are discussed with the ADMISSION Patient Advisory Group, who provide input on the design, interpretation and dissemination of results.

For peer review only - http://bmjopen.bmj.com/site/about/guidelines.xhtml

Analyses

We derived diagnoses made during, or at discharge, from ICD-10 codes recorded in the EHR over the 10 years prior to the index admission date. Code lists used for each diagnosis are given in Supplementary Material; the conditions selected were based on conditions previously used in UK BioBank, [9] modified to remove diagnoses less likely to be coded in hospital discharge data. Code lists were reviewed by clinicians in the research team prior to data extraction. We then examined the percentage of diagnoses that were captured by looking back from the date of index admission for increasing periods of time from 1 year to 10 years and compared the proportion of patients with diagnoses captured for each lookback period with the number of diagnoses captured with 10 years of lookback. Only individuals with a full 10 years of EHR data were included in the analyses. We chose a threshold of 90% completeness against which to report results; this threshold was agreed by clinician authors as representing a balance between accuracy and achievability.

Results

We included 560 individuals in the COPD exacerbation analysis dataset (32 were excluded due to incomplete lookback data), and 2142 individuals in the acute stroke dataset (189 were excluded due to incomplete lookback data). A flowchart depicting selection of patient admissions is shown in Figure 1. A total of 73 different diagnoses were recorded for those with COPD exacerbation (median 3 per individual) and a total of 80 different diagnoses were recorded for those with acute stroke (median 6 per individual). Table 1 shows details of individuals in each dataset, along with the top 10 most common diagnoses recorded in each dataset.

Figure 2 shows the percentage of each condition detected per year of lookback in the COPD dataset, using the number recorded over the full 10-year lookback period as reference. Seven of the top 10 reached 90% completeness by 6 years but two conditions (asthma and hypertension) continued to show increases in coding completeness with increasing duration of lookback through to 10 years.

For peer review only - http://bmjopen.bmj.com/site/about/guidelines.xhtml

Figure 3 shows similar data for the acute stroke dataset; there was considerable heterogeneity, from dementia (90% by 3 years) and myocardial infarction (90% by 4 years) through to diabetes mellitus (90% by 5 years) and angina pectoris (90% by 7 years). Previous transient ischaemic attack coding completeness continued to rise out to 10 years with no flattening of the curve.

Discussion

Both cohorts in our study had a high prevalence of MLTC, reinforcing the need for methodological studies in this area to ensure the robustness of analyses using routine data. Our results suggest that a seven year lookback is sufficient to capture some but not all common diagnoses for patients admitted with COPD exacerbations or acute stroke. For most diagnoses investigated, the prevalence curves flatten out between six and seven years, suggesting that additional years of lookback adds few additional diagnoses. There were however notable exceptions, particularly hypertension and asthma in the COPD cohort, and previous TIA in the acute stroke cohort. For these diagnoses, curves did not flatten at ten years, suggesting that even longer periods of lookback may be required to gain complete diagnostic data. These data suggest that the duration of lookback needs to be tailored to the condition of interest.

Our results give a useful indication of what the minimum lookback time in hospital electronic health records might need to be, but we are not able to ascertain why lookback time varies between diagnoses. Conditions which are the direct cause of hospital admission may be more likely to be recorded, and patients who have frequent hospital admissions may be more likely to have their associated diagnoses recorded. Conditions relevant to a particular speciality (for example respiratory diagnoses when admitted under a respiratory team) may be more likely to be recorded than those not associated with the admitted specialty. Further work is required to test these hypotheses however. For some diagnoses, reliance on coded, structured diagnostic data is likely to remain inadequate as

For peer review only - http://bmjopen.bmj.com/site/about/guidelines.xhtml

not all diagnoses are written down in hospital records, and for those that are, many are not recorded or coded in a structured manner. [10] It is these diagnoses that require additional work to extract, either from unstructured EHR data using techniques such as natural language processing, [11] by using additional data sources, for instance prescribing records [12] or laboratory data, [13] and by linkage to alternative sources of diagnostic data (for instance data from primary care). [14] Our results help to highlight diagnoses for which this work is particularly needed.

Strengths of our approach include the use of routinely collected electronic healthcare data and the interrogation of two common diagnostic reasons for hospital admission. Using routinely-collected electronic health data for MLTC research enables analyses to include a wide range of patients, many of whom are excluded from traditional cohort studies because of inability to consent due to illness or cognitive impairment, or who belong to other groups underserved by research such as socioeconomically disadvantaged groups. [15] Routine data also enables the use of very large sample sizes and provides granular longitudinal follow-up incorporating a wide range of conditions and outcomes. We were limited by an inability to go back further than 10 years without a reduction in the number of patients included in the analysis, and we have confined our analysis to the most common conditions associated with each index condition – other, less common conditions may require different lookback periods. Our choice of conditions was based on those previously analysed in UK Biobank, but UK Biobank does not use an exhaustive list of conditions and it is possible that other common and important conditions were not included in our analysis. Our choice of a 90% threshold for reporting analyses is to a degree arbitrary; different degrees of completeness may be appropriate for different analyses and only individual research or analysis teams can make a judgement as to what degree of completeness is appropriate for a particular analysis. Finally, our analysis is confined to a single hospital, and lookback times may vary across different healthcare organisations – organisations may vary in how well they code different diagnoses. Future research to replicate our work in different

For peer review only

hospitals and in different index conditions would be useful to increase the generalisability of our findings.

In summary, whilst a seven year lookback appears adequate to capture some common diagnoses associated with COPD exacerbation and acute stroke admissions, there is considerable variability between diagnoses. These findings will help both research teams and clinical teams decide what length of lookback they need to employ to ensure that capture of diagnoses are sufficiently complete for the intended purpose, whether research, clinical audit or as part of routine healthcare delivery. For electronic health records with only a short lookback, this may mean that certain diagnoses are not amenable to accurate interrogation. The variability in lookback suggests that to be sure that diagnoses of interest are captured with sufficient completeness, researchers need to evaluate such diagnoses in the specific EHR system that they use to be sure that diagnosis capture is suitably complete for the conditions they wish to study.

Funding

This research was conducted as part of the ADMISSION research collaborative, funded by the Strategic Priority Fund "Tackling multimorbidity at scale" programme [grant number MR/V033654/1]. This funding is delivered by the Medical Research Council and the National Institute for Health and Care Research in partnership with the Economic and Social Research Council and in collaboration with the Engineering and Physical Sciences Research Council. The views expressed in this publication are those of the authors and not necessarily those of UK Research and Innovation, the National Institute for Health and Care Research, or the Department of Health and Social Care.

Competing Interests

ES is director of the PIONEER acute data hub, funded by Health Data Research-UK. The other authors have no conflicts of interest to declare.

For peer review only - http://bmjopen.bmj.com/site/about/guidelines.xhtml

For peer review only

Author statement

JL: Conception and design; Data curation and analysis; Manuscript draft; critical revision

FE: Data curation and analysis; Interpretation of results; critical revision

RD: Data curation and analysis; critical revision

JF: Data curation and analysis; critical revision

SG: Data curation and analysis; critical revision

SH: Data curation and analysis; critical revision

PLR: Data curation and analysis; critical revision

MO: Data curation and analysis; critical revision

CP: Conception and design; Interpretation of results; critical revision

ES: Conception and design; Interpretation of results; critical revision

MS: Conception and design; Interpretation of results; critical revision

AAS: Conception and design; Interpretation of results; critical revision

MDW: Conception and design; Interpretation of results; Manuscript draft; critical revision

ADMISSION Research Collaborative: Conception, interpretation, critical revision

Ethics statement

This study was approved under umbrella ethics and Confidentiality Advisory Group (CAG) approvals which underpin the work of PIONEER, the Health Data Research (HDR)-UK Hub in Acute Care (East Midlands–Derby Research Ethics Committee reference: 20/EM/0158 and CAG reference: 20/CAG/0084). This Section 251 support via the NHS Health Research Authority Confidentiality Advisory Group gives permission for the use of routinely collected clinical data for research without individual participant consent.

Data statement

For peer review only

The data that support the findings of this study are not openly available due to reasons of sensitivity. Data may be accessed on request to the HDRUK PIONEER Acute Data Hub on provision of permission from the PIONEER Data Trust Committee and provision of a data access agreement. Data are located in controlled access data storage at the PIONEER acute data hub.

Acknowledgements

This work used data provided by patients and collected by the NHS as part of their care and support. MDW, CP and AAS acknowledge support from the National Institute for Health and Care Research (NIHR) Newcastle Biomedical Research Centre based at Newcastle upon Tyne Hospitals NHS Foundation Trust, Cumbria, Northumberland, Tyne and Wear NHS Foundation Trust and Newcastle University.

References

[1] Whitty CJ. Harveian Oration 2017: Triumphs and challenges in a world shaped by medicine. Clin Med (Lond) 2017;17:537-44.

[2] Chowdhury SR, Das DC, Sunna TC, Beyene J, Hossain A. Global and regional prevalence of multimorbidity in the adult population in community settings: a systematic review and meta-analysis. eClin Med 2023;57:101860.

[3] Barnett K, Mercer SW, Norbury M, Watt G, Wyke S, Guthrie B. Epidemiology of multimorbidity and implications for health care, research, and medical education: a cross-sectional study. Lancet 2012;380:37-43.

[4] Kudesia P, Salimarouny B, Stanley M, et al. The incidence of multimorbidity and patterns in accumulation of chronic conditions: A systematic review. J Comorb 2021;11:26335565211032880.

[5] Owen N, Dew L, Logan S, Denegri S, Chappell LC. Research policy for people with multiple long-term conditions and their carers. J Multimorb Comorb 2022;12:26335565221104407.

For peer review only

[6] Ugboma I, Syddall HE, Cox V, Cooper C, Briggs R, Sayer AA. Coding Geriatric syndromes: How good are we? CME J Geriatr Med 2008;10:34-6.

[7] Wright A, McCoy AB, Hickman TT, et al. Problem list completeness in electronic health records: A multi-site study and assessment of success factors. Int J Med Inform 2015;84:784-90.

[8] Gallier S, Price G, Pandya H, et al. Infrastructure and operating processes of PIONEER, the HDR-UK Data Hub in Acute Care and the workings of the Data Trust Committee: a protocol paper. BMJ Health Care Inform 2021;28:e100294.

[9] Gallacher KI, McQueenie R, Nicholl B, Jani BD, Lee D, Mair FS. Risk factors and mortality associated with multimorbidity in people with stroke or transient ischaemic attack: a study of 8,751 UK Biobank participants. J Comorb 2018;8:1– 8.

[10] Todd OM, Burton JK, Dodds RM, et al. New Horizons in the use of routine data for ageing research. Age Ageing 2020;49:716-22.

[11] Jackson RG, Patel R, Jayatilleke N, et al. Natural language processing to extract symptoms of severe mental illness from clinical text: the Clinical Record Interactive Search Comprehensive Data Extraction (CRIS-CODE) project. BMJ Open 2017;7:e012012.

[12] Hansen ER, Sagi T, Hose K, Lip GYH, Larsen TB, Skjøth F. Assigning diagnosis codes using medication history. Artif Intell Med 2022;128:102307.

[13] Schulz WL, Young P, Coppi A, et al. Temporal relationship of computed and structured diagnoses in electronic health record data. BMC Med Inform Decis Mak 2021;21:61.

[14] Herrett E, Shah AD, Boggon R, et al. Completeness and diagnostic validity of recording acute myocardial infarction events in primary care, hospital care, disease registry, and national mortality records: cohort study. BMJ 2013;346:f2350.

[15] Witham MD, Cooper R, Missier P, Robinson SM, Sapey E, Sayer AA. Researching multimorbidity in hospital: can we deliver on the promise of health informatics? Eur Geriatr Med 2023;14:765-8.

For peer review only - http://bmjopen.bmj.com/site/about/guidelines.xhtml

For peer review only

For peer review only - http://bmjopen.bmj.com/site/about/guidelines.xhtml

Table 1: Study Population details

	COPD dataset		Stroke dataset	
N	560		2142	
Mean age (years) (SD)	71 (11)		73 (13)	
Female sex (%)	296 (52)		1064 (50)	
Median number of comorbid conditions (IQR)*	3 (1 to 5)		6 (4 to 8)	
Top 10 previously diagnosed conditions**	Name	Frequency (%)	Name	Frequency (%)
1	COPD	560 (100)	Stroke	1729 (81)
2	Heart failure	227 (41)	Atrial fibrillation	843 (39)
3	Asthma	206 (37)	TIA	832 (39)
4	Atrial fibrillation	197 (35)	All Diabetes	804 (38)
5	Depression	182 (33)	Heart failure	631 (29)
6	Anxiety	180 (32)	COPD	525 (25)
7	All Diabetes	159 (28)	Myocardial infarction	519 (24)
8	Hypertension	151 (27)	Angina	491 (23)
9	Myocardial infarction	137 (24)	Dementia	432 (20)
10	Angina	126 (23)	Depression	426 (20)

*in addition to the index condition (COPD or stroke). **recorded at an admission prior to the index admission

For peer review only - http://bmjopen.bmj.com/site/about/guidelines.xhtml

For peer review only

Figure legends

Figure 1. Flow chart showing selection of patient episodes for inclusion in analysis

Figure 2. COPD exacerbation top 10 previously diagnosed conditions

Figure 3. Acute stroke top 10 previously diagnosed conditions

For peer review only

For peer review only - http://bmjopen.bmj.com/site/about/guidelines.xhtml

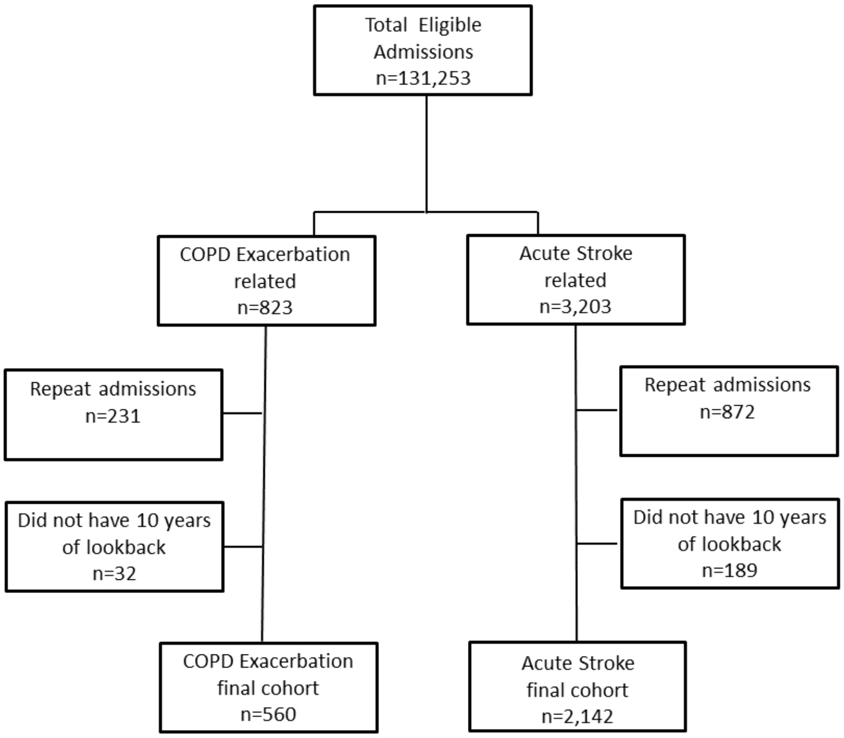

Flow chart showing selection of patient episodes for inclusion in analysis

641x481mm (38 x 38 DPI)

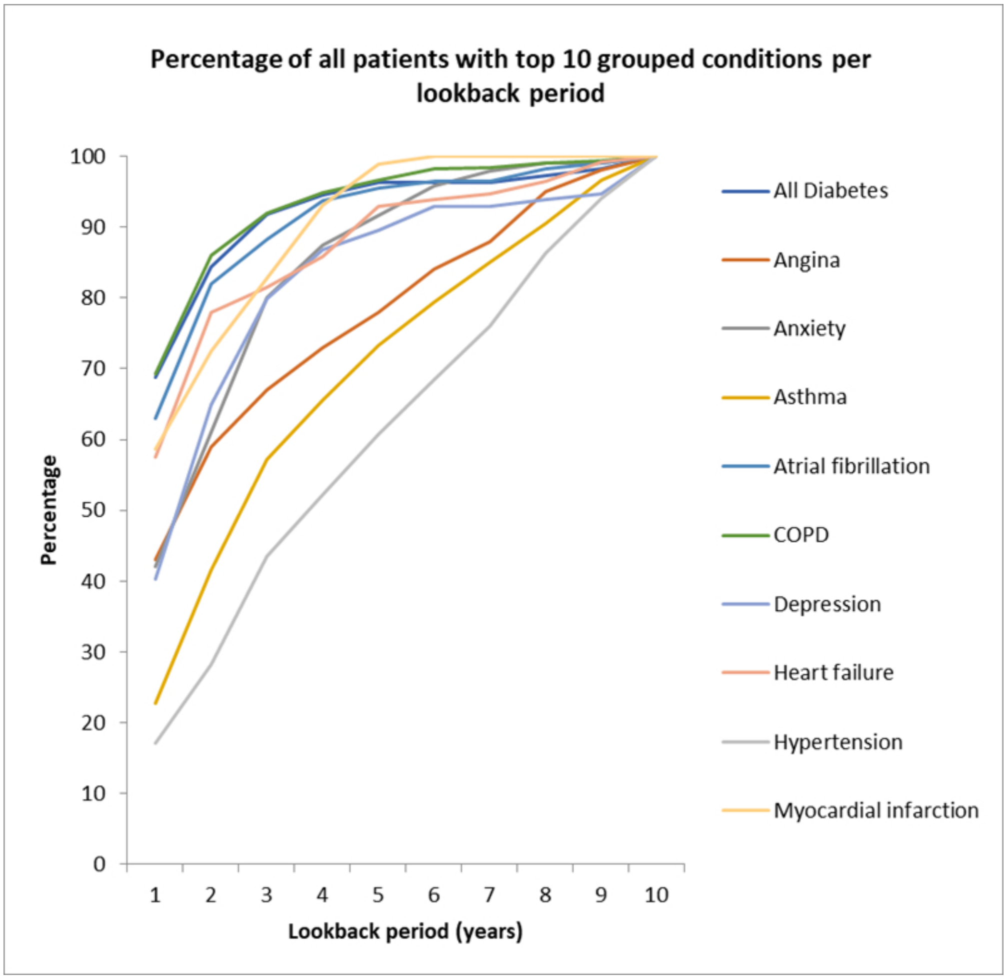

COPD exacerbation top 10 previously diagnosed conditions

343x333mm (59 x 59 DPI)

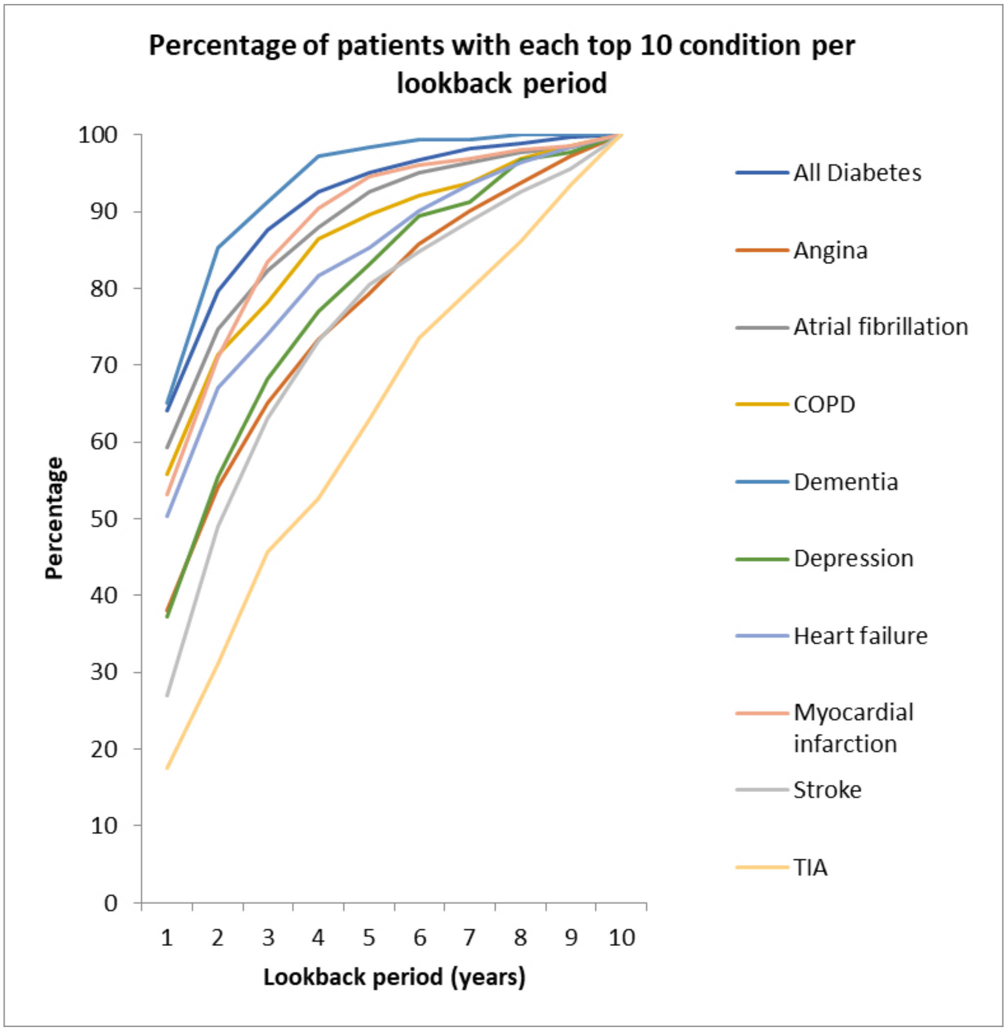

Acute stroke top 10 previously diagnosed conditions

338x346mm (59 x 59 DPI)

Supplementary Information

ADMISSION Research Collaborative Consortium members:

Avan Aihie Sayer[4,5]

Victoria Bartle[8]

Rachel Cooper[4,5]

Heather J Cordell[9]

Ray Holding[8]

Tom Marshall[10]

Fiona E Matthews[11]

Paolo Missier[12]

Ewan Pearson[13]

Chris Plummer[2,5]

Sian Robinson[4,5]

Elizabeth Sapey[1,6]

Mervyn Singer[7]

Thomas Scharf[11]

James Wason[14]

Miles D Witham[4,5]

1. PIONEER Hub, University of Birmingham, Birmingham, UK and Health Informatics, University Hospitals Birmingham NHS Foundation Trust, Birmingham, UK

2. Digital Services, Newcastle upon Tyne Hospitals NHS Foundation Trust, Newcastle upon Tyne, UK

4. AGE Research Group, Translational and Clinical Research Institute, Faculty of Medical Sciences, Newcastle University, Newcastle upon Tyne, UK

For peer review only

5. NIHR Newcastle Biomedical Research Centre, Newcastle upon Tyne Hospitals NHS Foundation Trust, Cumbria, Northumberland, Tyne and Wear NHS Foundation Trust and Newcastle University, Newcastle upon Tyne, UK

6. Institute of Inflammation and Ageing, University of Birmingham, Birmingham, UK.

7. University College London Hospitals NHS Foundation Trust, London, UK and Bloomsbury Institute for Intensive Care Medicine, London, UK

8. Public Co-Investigator, ADMISSION Research Collaborative, Newcastle upon Tyne, UK

9. Population Health Sciences Institute, Faculty of Medical Sciences, Newcastle University, Newcastle upon Tyne, UK

10. Institute of Applied Health Research, University of Birmingham, Birmingham, UK

11. Population Health Sciences Institute, Faculty of Medical Sciences, Newcastle University, Newcastle upon Tyne, UK

12. School of Computing, Newcastle University, Newcastle upon Tyne, UK

13. Division of Population Health and Genomics, Ninewells Hospital and School of Medicine, University of Dundee, Dundee, UK

14. Biostatistics Research Group, Population Health Sciences Institute, Newcastle University, Newcastle upon Tyne, UK

For peer review only

ICD-10 codes used to identify conditions

a. List of comorbid conditions with ICD-10 codes used to identify each condition

Stroke	I61, I63.0, I63.1, I63.2, I63.3, I63.4, I63.5, I63.8, I63.9, I69.1, I69.3, G46.3, G46.4, G46.5, G46.6, G46.7, G46.8, I64, I65, I69.4
Transient Ischaemic Attack	G45.0, G45.1, G45.2, G45.3, G45.4, G45.8, G45.9, G46.0, G46.1, G46.2, I65, I66
Subarachnoid haemorrhage	I60, I69.0
Carpal tunnel syndrome	G56.0
Fibromyalgia	M79.7
Disc problem	M50, M51
Prolapsed disc/slipped disc	M50, M51
Ankylosing spondylitis	M45
Osteoarthritis	M15, M16, M17, M18, M19
Gout	M10, M14.0
Trigeminal neuralgia	G50.0
Disc degeneration	M50, M51
Hypertension	I10, I11, I12, I13, I15
Depression	F32, F33
Asthma	J45, J46
Atrial fibrillation	I48
Myocardial infarction	I252, I21, I22, I23, I24.1
Angina	I20.0 I20.1, I20.8, I20.9
Gastro-oesophageal reflux	K21
Oesophagitis	K20, K21.0, K22.1
Barretts oesophagus	K22.7
Gastric ulcer	K25
Gastritis	K29
Duodenal ulcer	K26
Diabetes mellitus	E10, E11, E12, E13, E14, G59.0, G63.2, H28.0, H35.0, H36.0, M14.2, N08.3, O24.0, O24.1, O24.2, O24.3
Hyperthyroidism/thyrotoxicosis	E05.0, E05.1, E05.2, E05.5, E05.8, E05.9, E06.2
Hypothyroidism/myxoedema	E03.5, E03.8, E03.9
Systemic Lupus Erythematosus	L93, M32
Sjogrens syndrome/sicca syndrome	M35.0
Scleroderma/systemic sclerosis	M34
Rheumatoid arthritis	J99.0, M05, M06
Psoriatic arthropathy	M07.0, M07.1, M07.2, M07.3, M09.0, L40.5
Polymyalgia Rheumatica	M31.5, M35.3
Malabsorption/coeliac disease	K90.0
COPD	J40, J41, J42, J43, J44
Anxiety/panic attacks	F40, F41
Obsessive compulsive disorder	F42
Irritable bowel syndrome	K58
Alcohol dependency	I42.6

For peer review only

Alcoholic liver disease/alcoholic cirrhosis	K70
Renal/kidney failure	N18.5
Kidney nephropathy	N00.0
Diverticular disease	K38.2, K57
Diverticulitis	K38.2, K57
Peripheral vascular disease	I73.1, I73.8, I73.9, I74.3, I74.4, I74.5
Leg claudication/intermittent claudication	I73.1, I73.8, I73.9, I74.3, I74.4, I74.5
Cardiomyopathy	I42.0, I42.6, I25.5, I42.3, I42.5, I42.7, I42.8, I42.9, I43
Hypertrophic cardiomyopathy	I42.1, I42.2
Heart failure/pulmonary oedema	I11.0. I13.0, I13.2, I50
Prostate problem (not cancer)	N40
Enlarged prostate	N40
Benign prostatic hypertrophy	N40
Glaucoma	H40.1, H40.2, H40.9
Epilepsy	G40, G41
Dementia	F00, F01, F03, F05.1, G30, F02.3
Schizophrenia	F20, F21, F22, F25, F28, F29
Mania/bipolar disorder	F30, F31
Manic depression	F30, F31
Dermatitis	L20, L23, L24, L25, L26, L27, L28, L30.0, L30.1, L30.2, L30.5, L30.8, L30.9,
Psoriasis	L40, M07.0, M07.2, M07.3, M09.0
Crohn's disease	K50
Ulcerative colitis	K51
Migraine	G43
Chronic sinusitis	J32
Anorexia	F50.0, F50.1
Bulimia	F50.2, F50.3
Bronchiectasis	J47, Q33.4
Parkinson's disease	F02.3, G20
Multiple sclerosis	G35
Infective/viral hepatitis	B18
Hepatitis B	B18
Hepatitis C	B18
Hepatitis D	B18
Oesophageal varices	I85, I98.2, I98.3
Liver failure/cirrhosis	K70.1, K70.2, K70.3, K70.4, K71.1, K71.7, K72, K74.0, K74.1, K74.2, K74.4, K74.5, K74.6, K76.2, K76.3, B15.0, B16.0, B19.0
Osteoporosis	M80, M81, M82
Chronic fatigue syndrome	G93.3, F48.0
Endometriosis	N80
Meniere's disease	H81.0
Pernicious anaemia	D51
Polycystic ovary	E28.2

For peer review only

Lifetime diagnosis of cancer	Any C code, D00, D01, D02, D03, D04, D05, D06, D07, D08, D09, D49
Fragility fracture	S72.0, S72.1, S72.2, S52.5, S52.6, M48.5, M49.5
Incontinence	N39.3, N39.4
Delirium	F05

b. ICD-10 codes used to identify admissions for index conditions studied:

COPD exacerbation:

- J441,
- 195951007 (SNOMED),
- 196001008 (SNOMED)

Acute stroke:

- I63[subcodes 01234589],
- I693,
- I61
- I691,
- G46[subcodes 345678],
- I64
- I694
- G45[subcodes 0123489]
- G46[subcodes 012]
- I65
- I66

For peer review only

The RECORD statement – checklist of items, extended from the STROBE statement, that should be reported in observational studies using routinely collected health data.

	Item No.	STROBE items	Location in manuscript where items are reported	RECORD items	Location in manuscript where items are reported
**Title and abstract**					
	1	(a) Indicate the study's design with a commonly used term in the title or the abstract (b) Provide in the abstract an informative and balanced summary of what was done and what was found		RECORD 1.1: The type of data used should be specified in the title or abstract. When possible, the name of the databases used should be included.<br><br>RECORD 1.2: If applicable, the geographic region and timeframe within which the study took place should be reported in the title or abstract.<br><br>RECORD 1.3: If linkage between databases was conducted for the study, this should be clearly stated in the title or abstract.	Pages 1,3,4<br><br>Page 3-4<br><br>Page 3
**Introduction**					
Background rationale	2	Explain the scientific background and rationale for the investigation being reported			Page 5-6
Objectives	3	State specific objectives, including any prespecified hypotheses			Page 6
**Methods**					
Study Design	4	Present key elements of study design early in the paper			Page 6-8
Setting	5	Describe the setting, locations, and relevant dates, including periods of recruitment, exposure, follow-up, and data collection			Page 6-7

Participants	6	*(a) Cohort study* - Give the eligibility criteria, and the sources and methods of selection of participants. Describe methods of follow-up<br>*Case-control study* - Give the eligibility criteria, and the sources and methods of case ascertainment and control selection. Give the rationale for the choice of cases and controls<br>*Cross-sectional study* - Give the eligibility criteria, and the sources and methods of selection of participants<br><br>*(b) Cohort study* - For matched studies, give matching criteria and number of exposed and unexposed<br>*Case-control study* - For matched studies, give matching criteria and the number of controls per case		RECORD 6.1: The methods of study population selection (such as codes or algorithms used to identify subjects) should be listed in detail. If this is not possible, an explanation should be provided.<br><br>RECORD 6.2: Any validation studies of the codes or algorithms used to select the population should be referenced. If validation was conducted for this study and not published elsewhere, detailed methods and results should be provided.<br><br>RECORD 6.3: If the study involved linkage of databases, consider use of a flow diagram or other graphical display to demonstrate the data linkage process, including the number of individuals with linked data at each stage.	Supplementary material<br><br>N/A<br><br>Please see reference 8 (ref from page 7)
Variables	7	Clearly define all outcomes, exposures, predictors, potential confounders, and effect modifiers. Give diagnostic criteria, if applicable.		RECORD 7.1: A complete list of codes and algorithms used to classify exposures, outcomes, confounders, and effect modifiers should be provided. If these cannot be reported, an explanation should be provided.	Supplementary material
Data sources/ measurement	8	For each variable of interest, give sources of data and details of methods of assessment (measurement).<br>Describe comparability of assessment methods if there is more than one group			

For peer review only

Bias	9	Describe any efforts to address potential sources of bias			Page 6-8
Study size	10	Explain how the study size was arrived at			Page 6-7
Quantitative variables	11	Explain how quantitative variables were handled in the analyses. If applicable, describe which groupings were chosen, and why			Page 8
Statistical methods	12	(a) Describe all statistical methods, including those used to control for confounding<br>(b) Describe any methods used to examine subgroups and interactions<br>(c) Explain how missing data were addressed<br>(d) *Cohort study* - If applicable, explain how loss to follow-up was addressed<br>*Case-control study* - If applicable, explain how matching of cases and controls was addressed<br>*Cross-sectional study* - If applicable, describe analytical methods taking account of sampling strategy<br>(e) Describe any sensitivity analyses			Page 8<br>N/A<br>N/A<br>Page 8<br>N/A
Data access and cleaning methods		..		RECORD 12.1: Authors should describe the extent to which the investigators had access to the database population used to create the study population.	Please see reference 8

For peer review only

				RECORD 12.2: Authors should provide information on the data cleaning methods used in the study.	Please see reference 8
Linkage		..		RECORD 12.3: State whether the study included person-level, institutional-level, or other data linkage across two or more databases. The methods of linkage and methods of linkage quality evaluation should be provided.	Please see reference 8
**Results**					
Participants	13	(a) Report the numbers of individuals at each stage of the study (*e.g.*, numbers potentially eligible, examined for eligibility, confirmed eligible, included in the study, completing follow-up, and analysed)<br>(b) Give reasons for non-participation at each stage.<br>(c) Consider use of a flow diagram		RECORD 13.1: Describe in detail the selection of the persons included in the study (*i.e.,* study population selection) including filtering based on data quality, data availability and linkage. The selection of included persons can be described in the text and/or by means of the study flow diagram.	Figure 1
Descriptive data	14	(a) Give characteristics of study participants (*e.g.*, demographic, clinical, social) and information on exposures and potential confounders<br>(b) Indicate the number of participants with missing data for each variable of interest<br>(c) *Cohort study* - summarise follow-up time (*e.g.*, average and total amount)			Table 1
Outcome data	15	*Cohort study* - Report numbers of outcome events or summary measures over time<br>*Case-control study* - Report numbers in each exposure			Figures 2 and 3

For peer review only

		category, or summary measures of exposure<br>*Cross-sectional study* - Report numbers of outcome events or summary measures			
Main results	16	(a) Give unadjusted estimates and, if applicable, confounder-adjusted estimates and their precision (e.g., 95% confidence interval). Make clear which confounders were adjusted for and why they were included<br>(b) Report category boundaries when continuous variables were categorized<br>(c) If relevant, consider translating estimates of relative risk into absolute risk for a meaningful time period			Page 8-9
Other analyses	17	Report other analyses done—e.g., analyses of subgroups and interactions, and sensitivity analyses			N/A
**Discussion**					
Key results	18	Summarise key results with reference to study objectives			
Limitations	19	Discuss limitations of the study, taking into account sources of potential bias or imprecision. Discuss both direction and magnitude of any potential bias		RECORD 19.1: Discuss the implications of using data that were not created or collected to answer the specific research question(s). Include discussion of misclassification bias, unmeasured confounding, missing data, and changing eligibility over time, as they pertain to the study being reported.	Page 9
Interpretation	20	Give a cautious overall interpretation of results considering objectives,			Page 9-11

For peer review only

		limitations, multiplicity of analyses, results from similar studies, and other relevant evidence			
Generalisability	21	Discuss the generalisability (external validity) of the study results			Page 9-11
**Other Information**					
Funding	22	Give the source of funding and the role of the funders for the present study and, if applicable, for the original study on which the present article is based			Page 11
Accessibility of protocol, raw data, and programming code		..		RECORD 22.1: Authors should provide information on how to access any supplemental information such as the study protocol, raw data, or programming code.	Page 12

*Reference: Benchimol EI, Smeeth L, Guttmann A, Harron K, Moher D, Petersen I, Sørensen HT, von Elm E, Langan SM, the RECORD Working Committee. The REporting of studies Conducted using Observational Routinely-collected health Data (RECORD) Statement. *PLoS Medicine* 2015; in press.

*Checklist is protected under Creative Commons Attribution (CC BY) license.

For peer review only

BMJ Open

How far back do we need to look to capture diagnoses in electronic health records? An retrospective observational study of hospital electronic health record data

Journal:	*BMJ Open*
Manuscript ID	bmjopen-2023-080678.R2
Article Type:	Original research
Date Submitted by the Author:	30-Jan-2024
Complete List of Authors:	Lewis, Jadene; University of Birmingham, Health Informatics; University Hospitals Birmingham NHS Foundation Trust, Health Informatics<br>Evison, Felicity; University Hospitals Birmingham NHS Foundation Trust, Department of Health Informatics<br>Doal, Rominique; University of Birmingham, Health Informatics; University Hospitals Birmingham NHS Foundation Trust, Health Informatics<br>Field, Joanne; Newcastle Upon Tyne Hospitals NHS Foundation Trust<br>Gallier, Suzy; University Hospitals Birmingham NHS Foundation Trust, Health Informatics; University of Birmingham, PIONEER Technical Director<br>Harris, Steve; University College London Hospitals NHS Foundation Trust, Critical Care Department; University College London, Critical Care Department<br>le Roux, Peta; Newcastle Upon Tyne Hospitals NHS Foundation Trust<br>Osman, Mohammed; Newcastle University; Newcastle University<br>Plummer, Chris; Newcastle Upon Tyne Hospitals NHS Foundation Trust<br>Sapey, Elizabeth; University of Birmingham, Institute of Inflammation and Ageing<br>Singer, Mervyn; UCL, Bloomsbury Inst of Intensive Care Medicine<br>Sayer, Avan; Newcastle Upon Tyne Hospitals NHS Foundation Trust, NIHR Biomedical Research Centre; Newcastle University, Campus for Ageing and Vitality<br>Witham, Miles; Newcastle University; Newcastle University Research Collaborative, The ADMISSION; Newcastle University
**Primary Subject Heading**:	Research methods
Secondary Subject Heading:	Health informatics
Keywords:	Electronic Health Records, Information Extraction < Information Storage and Retrieval, Hospitals

SCHOLARONE™
Manuscripts

I, the Submitting Author has the right to grant and does grant on behalf of all authors of the Work (as defined in the below author licence), an exclusive licence and/or a non-exclusive licence for contributions from authors who are: i) UK Crown employees; ii) where BMJ has agreed a CC-BY licence shall apply, and/or iii) in accordance with the terms applicable for US Federal Government officers or employees acting as part of their official duties; on a worldwide, perpetual, irrevocable, royalty-free basis to BMJ Publishing Group Ltd ("BMJ") its licensees and where the relevant Journal is co-owned by BMJ to the co-owners of the Journal, to publish the Work in this journal and any other BMJ products and to exploit all rights, as set out in our licence.

The Submitting Author accepts and understands that any supply made under these terms is made by BMJ to the Submitting Author unless you are acting as an employee on behalf of your employer or a postgraduate student of an affiliated institution which is paying any applicable article publishing charge ("APC") for Open Access articles. Where the Submitting Author wishes to make the Work available on an Open Access basis (and intends to pay the relevant APC), the terms of reuse of such Open Access shall be governed by a Creative Commons licence – details of these licences and which Creative Commons licence will apply to this Work are set out in our licence referred to above.

Other than as permitted in any relevant BMJ Author's Self Archiving Policies, I confirm this Work has not been accepted for publication elsewhere, is not being considered for publication elsewhere and does not duplicate material already published. I confirm all authors consent to publication of this Work and authorise the granting of this licence.

For peer review only

How far back do we need to look to capture diagnoses in electronic health records? An retrospective observational study of hospital electronic health record data

Short title: Lookback in Queen Elizabeth Hospital Birmingham electronic health records

Jadene Lewis[1]

Felicity Evison[1]

Rominique Doal[1]

Joanne Field[2]

Suzy Gallier[1]

Steve Harris[3]

Peta le Roux[2]

Mohammed Osman[4,5]

Chris Plummer[2,5]

Elizabeth Sapey[1,6]

Mervyn Singer[7]

Avan Aihie Sayer[4,5]

Miles D Witham[4,5]

The ADMISSION Research Collaborative

1. PIONEER Hub, University of Birmingham, Birmingham, UK and Health Informatics, University Hospitals Birmingham NHS Foundation Trust, Birmingham, UK
2. Digital Services, Newcastle upon Tyne Hospitals NHS Foundation Trust, Newcastle upon Tyne, UK
3. Critical Care Department, University College London Hospitals NHS Foundation Trust, London, UK and Institute of Health Informatics, University College London, London, UK
4. AGE Research Group, Translational and Clinical Research Institute, Faculty of Medical Sciences, Newcastle University, Newcastle upon Tyne, UK
5. NIHR Newcastle Biomedical Research Centre, Newcastle upon Tyne Hospitals NHS Foundation Trust, Cumbria, Northumberland, Tyne and Wear NHS Foundation Trust and Newcastle University, Newcastle upon Tyne, UK
6. Institute of Inflammation and Ageing, University of Birmingham, Birmingham, UK.
7. University College London Hospitals NHS Foundation Trust, London, UK and Bloomsbury Institute for Intensive Care Medicine, London, UK

For peer review only - http://bmjopen.bmj.com/site/about/guidelines.xhtml

For peer review only

Correspondence to: Professor Miles Witham, AGE Research Group, NIHR Newcastle Biomedical Research Centre, 3rd floor, Biomedical Research Building, Campus for Ageing and Vitality, Newcastle upon Tyne, NE4 5PL. Tel: 0191 282 1317. Email: Miles.Witham@newcastle.ac.uk

Word count:

Abstract: 299 words

Main text: 1802

For peer review only

For peer review only - http://bmjopen.bmj.com/site/about/guidelines.xhtml

Abstract

Objectives:

Analysis of routinely-collected electronic health data is a key tool for long-term conditions research and practice for hospitalised patients. This requires accurate and complete ascertainment of a broad range of diagnoses; something not always recorded on an admission document at a single point in time. This study aimed to ascertain how far back in time electronic hospital records need to be interrogated to capture long-term condition diagnoses.

Design

Retrospective observational study of routinely collected hospital electronic health record data.

Setting

Queen Elizabeth Hospital Birmingham (UK) linked data held by the PIONEER acute care data hub.

Participants

Patients whose first recorded admission for COPD exacerbation (n=560) or acute stroke (n=2142) was between January and December 2018 and who had a minimum of 10 years of data prior to the index date.

Outcome measures

We identified the most common ICD-10 coded diagnoses received by COPD and acute stroke patients separately. For each diagnosis, we derived the number of patients with the diagnosis recorded at least once over the full 10-year lookback period, and then compared this with shorter lookback periods from 1 year to 9 years prior to the index admission.

For peer review only

Results

Seven of the top 10 commonest diagnoses in the COPD dataset reached >90% completeness by six years of lookback. Atrial fibrillation and diabetes were >90% coded with 2 to 3 years of lookback, but hypertension and asthma completeness continued to rise all the way out to 10 years of lookback. For stroke, 4 of the top 10 reached 90% completeness by 5 years of lookback; angina pectoris was >90% coded at 7 years and previous transient ischaemic attack completeness continued to rise out to 10 years of lookback.

Conclusion

A seven-year lookback captures most, but not all common diagnoses. Lookback duration should be tailored to the conditions being studied.

Strengths and limitations of this study

- We analysed common conditions associated with two different index conditions underlying hospital admission
- Our mature, large hospital dataset enabled us to study a variety of conditions and analyse longitudinal trends over ten years
- Less common associated conditions were not studied, and may require different lookback periods for effective capture
- Our analysis was confined to a single electronic hospital record and the required lookback periods could differ between hospitals

Keywords

Electronic health record; multimorbidity; multiple long-term conditions; diagnostic coding

For peer review only

Background

Multiple long-term conditions (MLTC) (multimorbidity) are an important but understudied challenge to healthcare systems. [1] MLTC, defined as two or more long-term health conditions, are common with an estimated global prevalence of 37%. [2] The prevalence in old age is even higher, reaching 80% by the age of 80 years in the UK. [3] Much of the research on MLTC to date has used large population-based datasets or has focussed on primary care data; there has been comparatively little work on MLTC within the hospital environment. [4] Understanding the associations, consequences, and pathways of care for MLTC within hospital is critical if we are to understand how best to prevent the adverse consequences of MLTC and deliver better care for hospitalised patients. [5]

Analysis of routinely-collected electronic healthcare records affords the opportunity to study MLTC at scale, across a wide range of patient groups. Use of routine data enables inclusion in analyses of those who would not be able to join traditional consented studies, such as those with dementia or with critical illness. The accurate identification of which long-term conditions affect hospitalised patients is central to the study of MLTC in patients admitted to hospital. In the UK, diagnoses within Electronic Healthcare Records (EHRs) are recorded at discharge, but can also be recorded during admissions. The quality and completeness of diagnostic coding is highly variable however, [6,7] and examining diagnoses coded during a single admission may not provide a complete picture of all diagnoses affecting an individual.

Aggregating diagnoses from a series of hospital admissions provides an opportunity to improve the completeness of recording, but it is unclear how far back through an electronic healthcare record researchers should look. Different EHRs have been in existence for different amounts of time, and comparison between EHRs may introduce bias if different lookback times introduce differences in the completeness of diagnostic recording. Understanding the completeness of diagnostic recording for a given lookback period would help to avoid under-recording of diagnoses in both research analyses and

For peer review only - http://bmjopen.bmj.com/site/about/guidelines.xhtml

clinical applications. This in turn would enable analysis teams to make informed judgements about what records and which conditions to study when analysing routinely-collected electronic health data.

The aim of this analysis was therefore to investigate how far back we need to look in order to capture the majority of diagnoses documented from previous admissions, and whether there are differences between diagnoses in what the minimum lookback period should be.

Methods

Hospital and data sources

The Queen Elizabeth Hospital Birmingham (QEHB) is a National Health Service acute hospital providing secondary and tertiary care services to adults in a large urban and suburban catchment area in England, UK. The hospital has 1269 beds including 80 level 2/3 intensive care beds and an Emergency Department that assesses >300 patients per day. QEHB uses an EHR (PICS, Birmingham Systems) containing time-stamped, structured records including demographics, location, time of admission and discharge. It also contains details of all treatments and investigations, and physiological measurements such as pulse, blood pressure and respiratory rate derived from the National Early Warning Score version 2 (NEWS2). Diagnoses are currently coded using the Systematised Nomenclature of Medicine – Clinical Terms (SNOMED-CT) system, and historically used International Classification of Diseases version 10 (ICD-10). The EHR has been in place since 1999 and the Trust is a paperless environment for all care provision and planning.

For this analysis, we used two data extracts. We extracted data on all acute admissions between 1st January 2018 and 31st December 2018 with a discharge diagnosis of COPD exacerbation (as a primary or secondary diagnosis). We also used SNOMED codes recorded in the electronic health record to resolve whether a diagnosis coded as 'COPD unspecified' was due to an acute exacerbation. Using this time period enabled us to avoid any distorting effects from the COVID-19 pandemic. We took the date

For peer review only - http://bmjopen.bmj.com/site/about/guidelines.xhtml

of the first hospital admission for COPD exacerbation in 2018 as the index date, and extracted data on age, sex and all hospital diagnoses coded by ICD-10 discharge codes within ten years prior to the admission under study. For the second data extract, we repeated the procedure but extracted data on all acute admissions with a discharge diagnosis of stroke. We chose COPD exacerbation and stroke as exemplar conditions as they are common causes of admission to hospital and are associated with a substantial burden of comorbidity.

Approval by PIONEER Data Trust Committee

This study was approved under umbrella ethics and Confidentiality Advisory Group (CAG) approvals which underpin the work of PIONEER, the Health Data Research (HDR)-UK Hub in Acute Care (East Midlands–Derby Research Ethics Committee reference: 20/EM/0158 and CAG reference: 20/CAG/0084). The NHS Health Research Authority Confidentiality Advisory Group gives permission for the use of routinely collected clinical data for research without individual participant consent, via section 251 of the National Health Service Act 2006. As part of this process, the data access request and specification were reviewed and approved by the PIONEER Data Trust Committee, comprising a group of patients and members of the public who provide oversight of all data access decisions within PIONEER. Governance and linkage processes for PIONEER data have been published previously. [8]

Patient and public involvement

The ADMISSION grant programme was co-designed with a panel of patients including patients with MLTC. Two public co-applicants form part of the management team for the ADMISSION programme, and studies within ADMISSION including this analysis are discussed with the ADMISSION Patient Advisory Group, who provide input on the design, interpretation and dissemination of results.

For peer review only

Analyses

We derived diagnoses made during, or at discharge, from ICD-10 codes recorded in the EHR over the 10 years prior to the index admission date. Code lists used for each diagnosis are given in Supplementary Material; the conditions selected were based on conditions previously used in UK BioBank, [9] modified to remove diagnoses less likely to be coded in hospital discharge data. Code lists were reviewed by clinicians in the research team prior to data extraction. We then examined the percentage of diagnoses that were captured by looking back from the date of index admission for increasing periods of time from 1 year to 10 years. We compared the proportion of patients with diagnoses captured for each lookback period with the number of diagnoses captured with 10 years of lookback. Only individuals with a full 10 years of EHR data were included in the analyses. We chose a threshold of 90% completeness against which to report results; this threshold was agreed by clinician authors as representing a balance between accuracy and achievability.

Results

We included 560 individuals in the COPD exacerbation analysis dataset (32 were excluded due to incomplete lookback data), and 2142 individuals in the acute stroke dataset (189 were excluded due to incomplete lookback data). A flowchart depicting selection of patient admissions is shown in Figure 1. A total of 73 different diagnoses were recorded for those with COPD exacerbation (median 3 per individual) and a total of 80 different diagnoses were recorded for those with acute stroke (median 6 per individual). Table 1 shows details of individuals in each dataset, along with the top 10 most common diagnoses recorded in each dataset.

Figure 2 shows the percentage of each condition detected per year of lookback in the COPD dataset, using the number recorded over the full 10-year lookback period as reference. Seven of the top 10 reached 90% completeness by 6 years but two conditions (asthma and hypertension) continued to show increases in coding completeness with increasing duration of lookback through to 10 years.

For peer review only - http://bmjopen.bmj.com/site/about/guidelines.xhtml

Figure 3 shows similar data for the acute stroke dataset; there was considerable heterogeneity, from dementia (90% by 3 years) and myocardial infarction (90% by 4 years) through to diabetes mellitus (90% by 5 years) and angina pectoris (90% by 7 years). Previous transient ischaemic attack coding completeness continued to increase through to 10 years with no flattening of the curve.

Discussion

Both cohorts in our study had a high prevalence of MLTC, reinforcing the need for methodological studies in this area to ensure the robustness of analyses using routine data. Our results suggest that a seven year lookback is sufficient to capture some but not all common diagnoses for patients admitted with COPD exacerbations or acute stroke. For most diagnoses investigated, the prevalence curves flatten out between six and seven years, suggesting that additional years of lookback add few additional diagnoses. There were however notable exceptions, particularly hypertension and asthma in the COPD cohort, and previous TIA in the acute stroke cohort. For these diagnoses, the curves did not flatten at ten years, suggesting that even longer periods of lookback may be required to gain complete diagnostic data. These data suggest that the duration of lookback needs to be tailored to the condition of interest.

Our results give a useful indication of what the minimum lookback time in hospital electronic health records might need to be, but we are not able to ascertain why lookback time varies between diagnoses. Conditions which are the direct cause of hospital admission may be more likely to be recorded, and patients who have frequent hospital admissions may be more likely to have their associated diagnoses recorded. Conditions relevant to a particular speciality (for example respiratory diagnoses when admitted under a respiratory team) may be more likely to be recorded than those not associated with the admitting specialty. Further work is required to test these hypotheses. For some diagnoses, reliance on coded, structured diagnostic data is likely to remain inadequate as not all diagnoses are written down in hospital records. Even for those diagnoses that are written down, many

For peer review only

For peer review only - http://bmjopen.bmj.com/site/about/guidelines.xhtml

are not recorded or coded in a structured manner. [10] Extracting these diagnoses requires additional work, for instance by interrogating unstructured EHR data using techniques such as natural language processing, [11] by using additional data sources, for instance prescribing records [12] or laboratory data, [13] and by linkage to alternative sources of diagnostic data (for instance data from primary care). [14] Our results help to highlight diagnoses for which this work is particularly needed.

Strengths of our approach include the use of routinely collected electronic healthcare data and the interrogation of two common diagnostic reasons for hospital admission. Using routinely-collected electronic health data for MLTC research enables inclusion of a wide range of patients. This includes groups excluded from traditional cohort studies because of inability to consent due to illness or cognitive impairment, or who belong to other groups underserved by research such as socioeconomically disadvantaged groups. [15]

Routine data also enables the use of very large sample sizes and can provide detailed longitudinal follow-up incorporating a wide range of conditions and outcomes. We were unable to look back further than 10 years without a reduction in the number of patients included in the analysis, and we have confined our analysis to the most common conditions associated with each index condition. Other less common conditions may require different lookback periods. Our choice of conditions was based on those previously analysed in UK Biobank, but UK Biobank does not use an exhaustive list of conditions and it is possible that other common and important conditions were not included in our analysis. Our choice of a 90% threshold for reporting analyses is to a degree arbitrary; different degrees of completeness may be appropriate for different analyses and only individual research or analysis teams can make a judgement as to what degree of completeness is appropriate for a particular analysis. Finally, our analysis is confined to a single hospital, and lookback times may vary across different healthcare organisations, who may vary in how well they code different diagnoses.

For peer review only - http://bmjopen.bmj.com/site/about/guidelines.xhtml

For peer review only

Future research to replicate our work in different hospitals and in different index conditions would be useful to increase the generalisability of our findings.

In summary, whilst a seven year lookback appears adequate to capture some common diagnoses associated with COPD exacerbation and acute stroke admissions, there is considerable variability between diagnoses. These findings will help both research teams and clinical teams decide what length of lookback they need to employ to ensure that capture of diagnoses are sufficiently complete for the intended purpose, whether this is for research, clinical audit or as part of routine healthcare delivery. For electronic health records with only a short lookback window, this may mean that certain diagnoses are not amenable to accurate interrogation. The variability in lookback suggests that researchers need to evaluate diagnoses in the specific EHR system that they use to be sure that diagnosis capture is suitably complete for the conditions they wish to study.

Funding

This research was conducted as part of the ADMISSION research collaborative, funded by the Strategic Priority Fund “Tackling multimorbidity at scale” programme [grant number MR/V033654/1]. This funding is delivered by the Medical Research Council and the National Institute for Health and Care Research in partnership with the Economic and Social Research Council and in collaboration with the Engineering and Physical Sciences Research Council. The views expressed in this publication are those of the authors and not necessarily those of UK Research and Innovation, the National Institute for Health and Care Research, or the Department of Health and Social Care.

Competing Interests

ES is director of the PIONEER acute data hub, funded by Health Data Research-UK. The other authors have no conflicts of interest to declare.

For peer review only - http://bmjopen.bmj.com/site/about/guidelines.xhtml

Author statement

JL: Conception and design; Data curation and analysis; Manuscript draft; critical revision

FE: Data curation and analysis; Interpretation of results; critical revision

RD: Data curation and analysis; critical revision

JF: Data curation and analysis; critical revision

SG: Data curation and analysis; critical revision

SH: Data curation and analysis; critical revision

PLR: Data curation and analysis; critical revision

MO: Data curation and analysis; critical revision

CP: Conception and design; Interpretation of results; critical revision

ES: Conception and design; Interpretation of results; critical revision

MS: Conception and design; Interpretation of results; critical revision

AAS: Conception and design; Interpretation of results; critical revision

MDW: Conception and design; Interpretation of results; Manuscript draft; critical revision

ADMISSION Research Collaborative: Conception, interpretation, critical revision

Ethics statement

This study was approved under umbrella ethics and Confidentiality Advisory Group (CAG) approvals which underpin the work of PIONEER, the Health Data Research (HDR)-UK Hub in Acute Care (East Midlands–Derby Research Ethics Committee reference: 20/EM/0158 and CAG reference: 20/CAG/0084). This Section 251 support via the NHS Health Research Authority Confidentiality Advisory Group gives permission for the use of routinely collected clinical data for research without individual participant consent.

Data statement

For peer review only - http://bmjopen.bmj.com/site/about/guidelines.xhtml

The data that support the findings of this study are not openly available due to reasons of sensitivity. Data may be accessed on request to the HDRUK PIONEER Acute Data Hub on provision of permission from the PIONEER Data Trust Committee and provision of a data access agreement. Data are located in controlled access data storage at the PIONEER acute data hub.

Acknowledgements

This work used data provided by patients and collected by the NHS as part of their care and support. MDW, CP and AAS acknowledge support from the National Institute for Health and Care Research (NIHR) Newcastle Biomedical Research Centre based at Newcastle upon Tyne Hospitals NHS Foundation Trust, Cumbria, Northumberland, Tyne and Wear NHS Foundation Trust and Newcastle University.

References

[1] Whitty CJ. Harveian Oration 2017: Triumphs and challenges in a world shaped by medicine. Clin Med (Lond) 2017;17:537-44.

[2] Chowdhury SR, Das DC, Sunna TC, Beyene J, Hossain A. Global and regional prevalence of multimorbidity in the adult population in community settings: a systematic review and meta-analysis. eClin Med 2023;57:101860.

[3] Barnett K, Mercer SW, Norbury M, Watt G, Wyke S, Guthrie B. Epidemiology of multimorbidity and implications for health care, research, and medical education: a cross-sectional study. Lancet 2012;380:37-43.

[4] Kudesia P, Salimarouny B, Stanley M, et al. The incidence of multimorbidity and patterns in accumulation of chronic conditions: A systematic review. J Comorb 2021;11:26335565211032880.

[5] Owen N, Dew L, Logan S, Denegri S, Chappell LC. Research policy for people with multiple long-term conditions and their carers. J Multimorb Comorb 2022;12:26335565221104407.

For peer review only - http://bmjopen.bmj.com/site/about/guidelines.xhtml

[6] Ugboma I, Syddall HE, Cox V, Cooper C, Briggs R, Sayer AA. Coding Geriatric syndromes: How good are we? CME J Geriatr Med 2008;10:34-6.

[7] Wright A, McCoy AB, Hickman TT, et al. Problem list completeness in electronic health records: A multi-site study and assessment of success factors. Int J Med Inform 2015;84:784-90.

[8] Gallier S, Price G, Pandya H, et al. Infrastructure and operating processes of PIONEER, the HDR-UK Data Hub in Acute Care and the workings of the Data Trust Committee: a protocol paper. BMJ Health Care Inform 2021;28:e100294.

[9] Gallacher KI, McQueenie R, Nicholl B, Jani BD, Lee D, Mair FS. Risk factors and mortality associated with multimorbidity in people with stroke or transient ischaemic attack: a study of 8,751 UK Biobank participants. J Comorb 2018;8:1– 8.

[10] Todd OM, Burton JK, Dodds RM, et al. New Horizons in the use of routine data for ageing research. Age Ageing 2020;49:716-22.

[11] Jackson RG, Patel R, Jayatilleke N, et al. Natural language processing to extract symptoms of severe mental illness from clinical text: the Clinical Record Interactive Search Comprehensive Data Extraction (CRIS-CODE) project. BMJ Open 2017;7:e012012.

[12] Hansen ER, Sagi T, Hose K, Lip GYH, Larsen TB, Skjøth F. Assigning diagnosis codes using medication history. Artif Intell Med 2022;128:102307.

[13] Schulz WL, Young P, Coppi A, et al. Temporal relationship of computed and structured diagnoses in electronic health record data. BMC Med Inform Decis Mak 2021;21:61.

[14] Herrett E, Shah AD, Boggon R, et al. Completeness and diagnostic validity of recording acute myocardial infarction events in primary care, hospital care, disease registry, and national mortality records: cohort study. BMJ 2013;346:f2350.

[15] Witham MD, Cooper R, Missier P, Robinson SM, Sapey E, Sayer AA. Researching multimorbidity in hospital: can we deliver on the promise of health informatics? Eur Geriatr Med 2023;14:765-8.

For peer review only

For peer review only - http://bmjopen.bmj.com/site/about/guidelines.xhtml

Table 1: Study Population details

	COPD dataset		Stroke dataset	
N	560		2142	
Mean age (years) (SD)	71 (11)		73 (13)	
Female sex (%)	296 (52)		1064 (50)	
Median number of comorbid conditions (IQR)*	3 (1 to 5)		6 (4 to 8)	
Top 10 previously diagnosed conditions**	Name	Frequency (%)	Name	Frequency (%)
1	COPD	560 (100)	Stroke	1729 (81)
2	Heart failure	227 (41)	Atrial fibrillation	843 (39)
3	Asthma	206 (37)	TIA	832 (39)
4	Atrial fibrillation	197 (35)	All Diabetes	804 (38)
5	Depression	182 (33)	Heart failure	631 (29)
6	Anxiety	180 (32)	COPD	525 (25)
7	All Diabetes	159 (28)	Myocardial infarction	519 (24)
8	Hypertension	151 (27)	Angina	491 (23)
9	Myocardial infarction	137 (24)	Dementia	432 (20)
10	Angina	126 (23)	Depression	426 (20)

*in addition to the index condition (COPD or stroke). **recorded at an admission prior to the index admission

For peer review only

For peer review only - http://bmjopen.bmj.com/site/about/guidelines.xhtml

Figure legends

Figure 1. Flow chart showing selection of patient episodes for inclusion in analysis

Figure 2. COPD exacerbation top 10 previously diagnosed conditions

Figure 3. Acute stroke top 10 previously diagnosed conditions

For peer review only

For peer review only - http://bmjopen.bmj.com/site/about/guidelines.xhtml

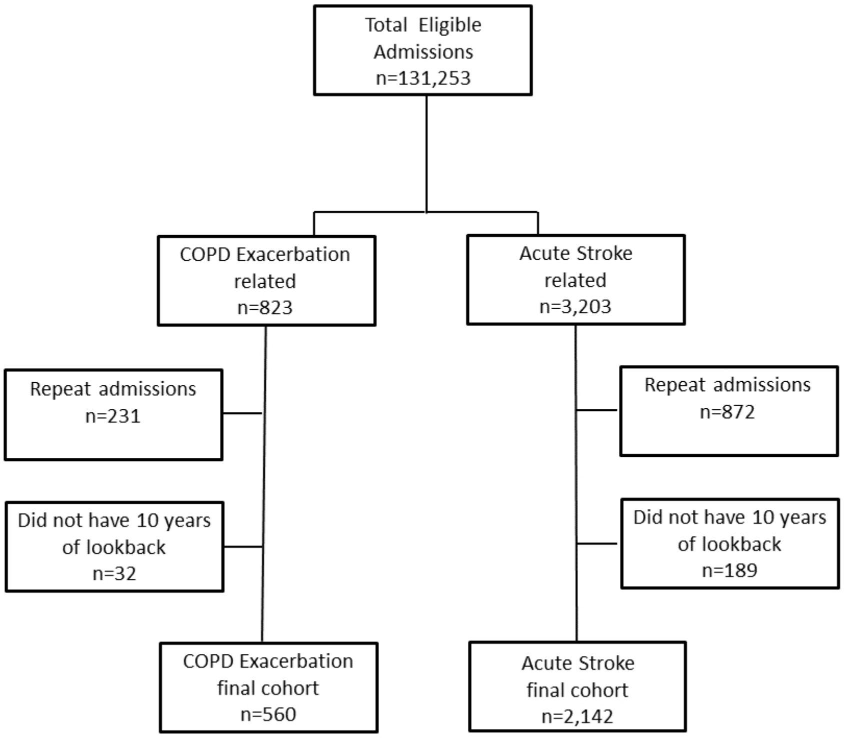

Flow chart showing selection of patient episodes for inclusion in analysis

641x481mm (38 x 38 DPI)

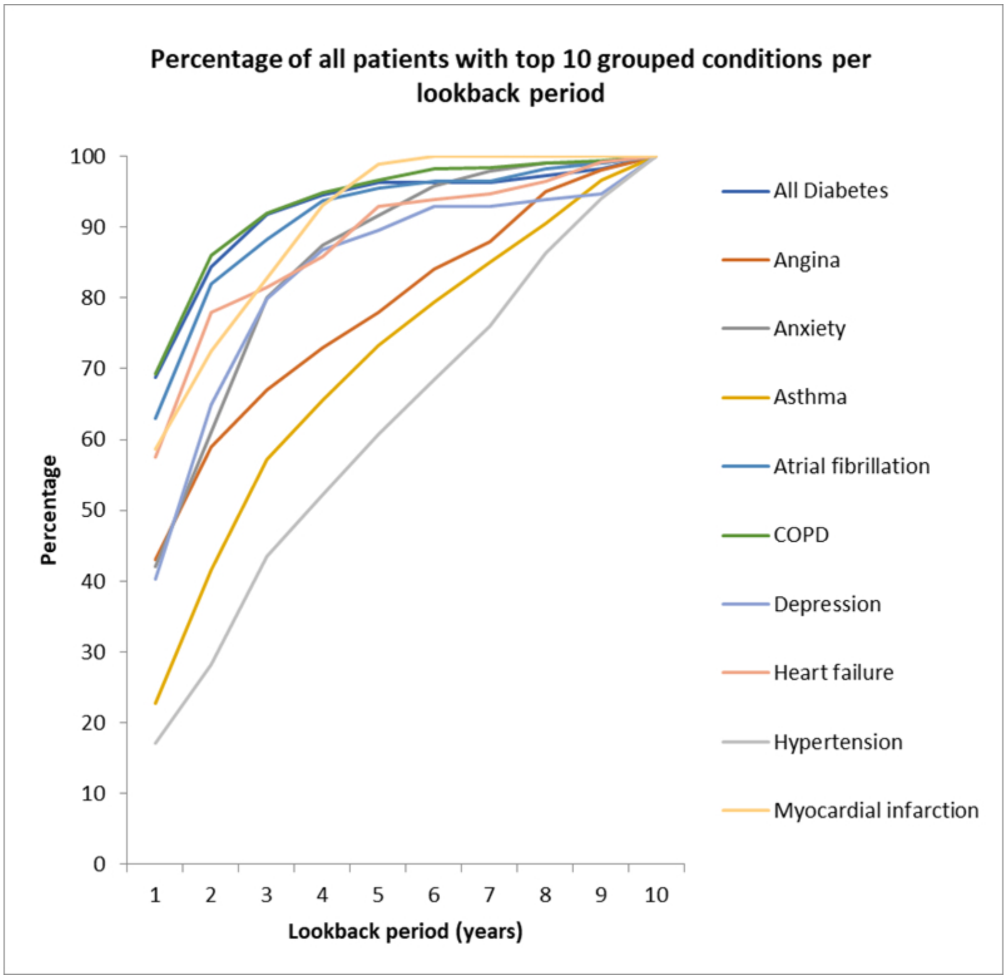

COPD exacerbation top 10 previously diagnosed conditions

343x333mm (59 x 59 DPI)

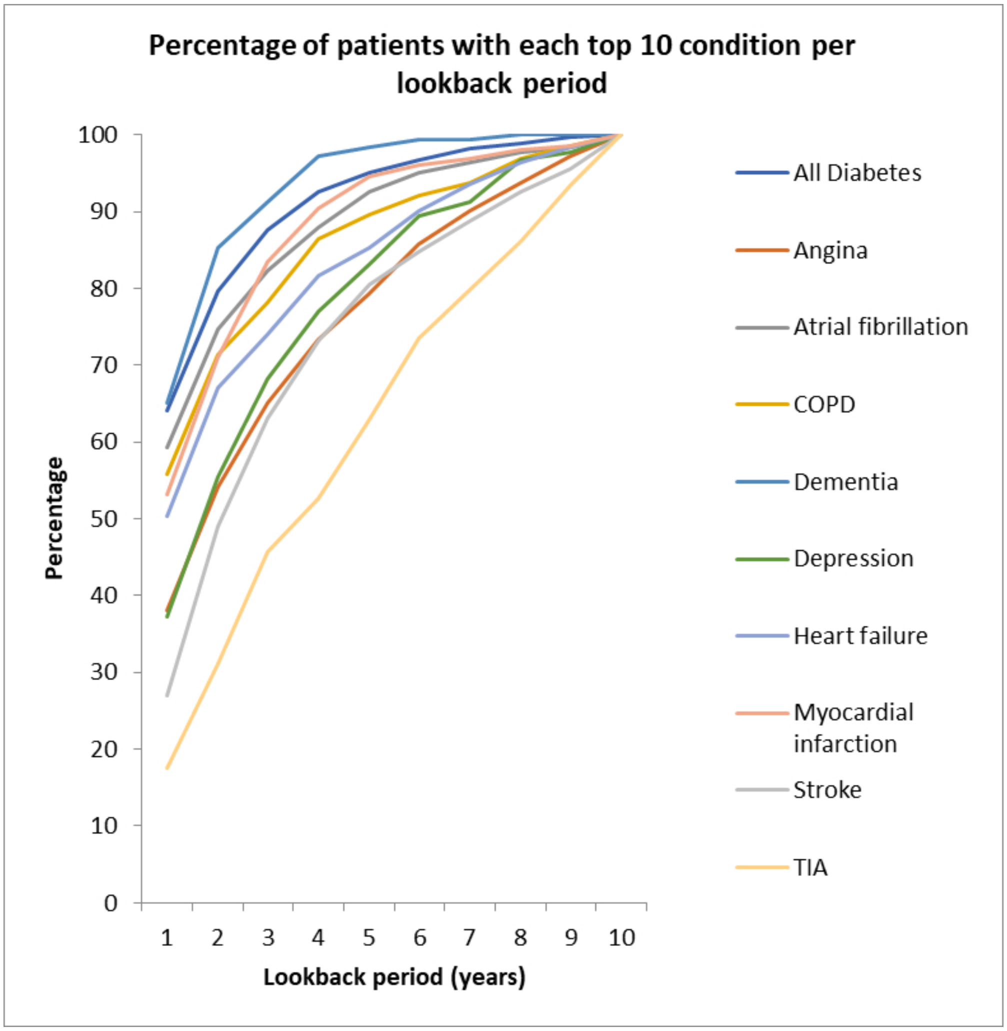

Acute stroke top 10 previously diagnosed conditions

338x346mm (59 x 59 DPI)

Supplementary Information

ADMISSION Research Collaborative Consortium members:

Avan Aihie Sayer[4,5]

Victoria Bartle[8]

Rachel Cooper[4,5]

Heather J Cordell[9]

Ray Holding[8]

Tom Marshall[10]

Fiona E Matthews[11]

Paolo Missier[12]

Ewan Pearson[13]

Chris Plummer[2,5]

Sian Robinson[4,5]

Elizabeth Sapey[1,6]

Mervyn Singer[7]

Thomas Scharf[11]

James Wason[14]

Miles D Witham[4,5]

1. PIONEER Hub, University of Birmingham, Birmingham, UK and Health Informatics, University Hospitals Birmingham NHS Foundation Trust, Birmingham, UK

2. Digital Services, Newcastle upon Tyne Hospitals NHS Foundation Trust, Newcastle upon Tyne, UK

4. AGE Research Group, Translational and Clinical Research Institute, Faculty of Medical Sciences, Newcastle University, Newcastle upon Tyne, UK

For peer review only

5. NIHR Newcastle Biomedical Research Centre, Newcastle upon Tyne Hospitals NHS Foundation Trust, Cumbria, Northumberland, Tyne and Wear NHS Foundation Trust and Newcastle University, Newcastle upon Tyne, UK

6. Institute of Inflammation and Ageing, University of Birmingham, Birmingham, UK.

7. University College London Hospitals NHS Foundation Trust, London, UK and Bloomsbury Institute for Intensive Care Medicine, London, UK

8. Public Co-Investigator, ADMISSION Research Collaborative, Newcastle upon Tyne, UK

9. Population Health Sciences Institute, Faculty of Medical Sciences, Newcastle University, Newcastle upon Tyne, UK

10. Institute of Applied Health Research, University of Birmingham, Birmingham, UK

11. Population Health Sciences Institute, Faculty of Medical Sciences, Newcastle University, Newcastle upon Tyne, UK

12. School of Computing, Newcastle University, Newcastle upon Tyne, UK

13. Division of Population Health and Genomics, Ninewells Hospital and School of Medicine, University of Dundee, Dundee, UK

14. Biostatistics Research Group, Population Health Sciences Institute, Newcastle University, Newcastle upon Tyne, UK

For peer review only

ICD-10 codes used to identify conditions

a. List of comorbid conditions with ICD-10 codes used to identify each condition

Stroke	I61, I63.0, I63.1, I63.2, I63.3, I63.4, I63.5, I63.8, I63.9, I69.1, I69.3, G46.3, G46.4, G46.5, G46.6, G46.7, G46.8, I64, I65, I69.4
Transient Ischaemic Attack	G45.0, G45.1, G45.2, G45.3, G45.4, G45.8, G45.9, G46.0, G46.1, G46.2, I65, I66
Subarachnoid haemorrhage	I60, I69.0
Carpal tunnel syndrome	G56.0
Fibromyalgia	M79.7
Disc problem	M50, M51
Prolapsed disc/slipped disc	M50, M51
Ankylosing spondylitis	M45
Osteoarthritis	M15, M16, M17, M18, M19
Gout	M10, M14.0
Trigeminal neuralgia	G50.0
Disc degeneration	M50, M51
Hypertension	I10, I11, I12, I13, I15
Depression	F32, F33
Asthma	J45, J46
Atrial fibrillation	I48
Myocardial infarction	I252, I21, I22, I23, I24.1
Angina	I20.0 I20.1, I20.8, I20.9
Gastro-oesophageal reflux	K21
Oesophagitis	K20, K21.0, K22.1
Barretts oesophagus	K22.7
Gastric ulcer	K25
Gastritis	K29
Duodenal ulcer	K26
Diabetes mellitus	E10, E11, E12, E13, E14, G59.0, G63.2, H28.0, H35.0, H36.0, M14.2, N08.3, O24.0, O24.1, O24.2, O24.3
Hyperthyroidism/thyrotoxicosis	E05.0, E05.1, E05.2, E05.5, E05.8, E05.9, E06.2
Hypothyroidism/myxoedema	E03.5, E03.8, E03.9
Systemic Lupus Erythematosus	L93, M32
Sjogrens syndrome/sicca syndrome	M35.0
Scleroderma/systemic sclerosis	M34
Rheumatoid arthritis	J99.0, M05, M06
Psoriatic arthropathy	M07.0, M07.1, M07.2, M07.3, M09.0, L40.5
Polymyalgia Rheumatica	M31.5, M35.3
Malabsorption/coeliac disease	K90.0
COPD	J40, J41, J42, J43, J44
Anxiety/panic attacks	F40, F41
Obsessive compulsive disorder	F42
Irritable bowel syndrome	K58
Alcohol dependency	I42.6

For peer review only

Alcoholic liver disease/alcoholic cirrhosis	K70
Renal/kidney failure	N18.5
Kidney nephropathy	N00.0
Diverticular disease	K38.2, K57
Diverticulitis	K38.2, K57
Peripheral vascular disease	I73.1, I73.8, I73.9, I74.3, I74.4, I74.5
Leg claudication/intermittent claudication	I73.1, I73.8, I73.9, I74.3, I74.4, I74.5
Cardiomyopathy	I42.0, I42.6, I25.5, I42.3, I42.5, I42.7, I42.8, I42.9, I43
Hypertrophic cardiomyopathy	I42.1, I42.2
Heart failure/pulmonary oedema	I11.0. I13.0, I13.2, I50
Prostate problem (not cancer)	N40
Enlarged prostate	N40
Benign prostatic hypertrophy	N40
Glaucoma	H40.1, H40.2, H40.9
Epilepsy	G40, G41
Dementia	F00, F01, F03, F05.1, G30, F02.3
Schizophrenia	F20, F21, F22, F25, F28, F29
Mania/bipolar disorder	F30, F31
Manic depression	F30, F31
Dermatitis	L20, L23, L24, L25, L26, L27, L28, L30.0, L30.1, L30.2, L30.5, L30.8, L30.9,
Psoriasis	L40, M07.0, M07.2, M07.3, M09.0
Crohn's disease	K50
Ulcerative colitis	K51
Migraine	G43
Chronic sinusitis	J32
Anorexia	F50.0, F50.1
Bulimia	F50.2, F50.3
Bronchiectasis	J47, Q33.4
Parkinson's disease	F02.3, G20
Multiple sclerosis	G35
Infective/viral hepatitis	B18
Hepatitis B	B18
Hepatitis C	B18
Hepatitis D	B18
Oesophageal varices	I85, I98.2, I98.3
Liver failure/cirrhosis	K70.1, K70.2, K70.3, K70.4, K71.1, K71.7, K72, K74.0, K74.1, K74.2, K74.4, K74.5, K74.6, K76.2, K76.3, B15.0, B16.0, B19.0
Osteoporosis	M80, M81, M82
Chronic fatigue syndrome	G93.3, F48.0
Endometriosis	N80
Meniere's disease	H81.0
Pernicious anaemia	D51
Polycystic ovary	E28.2

For peer review only

Lifetime diagnosis of cancer	Any C code, D00, D01, D02, D03, D04, D05, D06, D07, D08, D09, D49
Fragility fracture	S72.0, S72.1, S72.2, S52.5, S52.6, M48.5, M49.5
Incontinence	N39.3, N39.4
Delirium	F05

b. ICD-10 codes used to identify admissions for index conditions studied:

COPD exacerbation:

- J441,
- 195951007 (SNOMED),
- 196001008 (SNOMED)

Acute stroke:

- I63[subcodes 01234589],
- I693,
- I61
- I691,
- G46[subcodes 345678],
- I64
- I694
- G45[subcodes 0123489]
- G46[subcodes 012]
- I65
- I66

For peer review only

The RECORD statement – checklist of items, extended from the STROBE statement, that should be reported in observational studies using routinely collected health data.

	Item No.	STROBE items	Location in manuscript where items are reported	RECORD items	Location in manuscript where items are reported
**Title and abstract**					
	1	(a) Indicate the study's design with a commonly used term in the title or the abstract (b) Provide in the abstract an informative and balanced summary of what was done and what was found		RECORD 1.1: The type of data used should be specified in the title or abstract. When possible, the name of the databases used should be included.<br><br>RECORD 1.2: If applicable, the geographic region and timeframe within which the study took place should be reported in the title or abstract.<br><br>RECORD 1.3: If linkage between databases was conducted for the study, this should be clearly stated in the title or abstract.	Pages 1,3,4<br><br>Page 3-4<br><br>Page 3
**Introduction**					
Background rationale	2	Explain the scientific background and rationale for the investigation being reported			Page 5-6
Objectives	3	State specific objectives, including any prespecified hypotheses			Page 6
**Methods**					
Study Design	4	Present key elements of study design early in the paper			Page 6-8
Setting	5	Describe the setting, locations, and relevant dates, including periods of recruitment, exposure, follow-up, and data collection			Page 6-7

For peer review only

Participants	6	*(a) Cohort study* - Give the eligibility criteria, and the sources and methods of selection of participants. Describe methods of follow-up<br>*Case-control study* - Give the eligibility criteria, and the sources and methods of case ascertainment and control selection. Give the rationale for the choice of cases and controls<br>*Cross-sectional study* - Give the eligibility criteria, and the sources and methods of selection of participants<br><br>*(b) Cohort study* - For matched studies, give matching criteria and number of exposed and unexposed<br>*Case-control study* - For matched studies, give matching criteria and the number of controls per case		RECORD 6.1: The methods of study population selection (such as codes or algorithms used to identify subjects) should be listed in detail. If this is not possible, an explanation should be provided.<br><br>RECORD 6.2: Any validation studies of the codes or algorithms used to select the population should be referenced. If validation was conducted for this study and not published elsewhere, detailed methods and results should be provided.<br><br>RECORD 6.3: If the study involved linkage of databases, consider use of a flow diagram or other graphical display to demonstrate the data linkage process, including the number of individuals with linked data at each stage.	Supplementary material<br><br>N/A<br><br>Please see reference 8 (ref from page 7)
Variables	7	Clearly define all outcomes, exposures, predictors, potential confounders, and effect modifiers. Give diagnostic criteria, if applicable.		RECORD 7.1: A complete list of codes and algorithms used to classify exposures, outcomes, confounders, and effect modifiers should be provided. If these cannot be reported, an explanation should be provided.	Supplementary material
Data sources/ measurement	8	For each variable of interest, give sources of data and details of methods of assessment (measurement).<br>Describe comparability of assessment methods if there is more than one group			

For peer review only

Bias	9	Describe any efforts to address potential sources of bias			Page 6-8
Study size	10	Explain how the study size was arrived at			Page 6-7
Quantitative variables	11	Explain how quantitative variables were handled in the analyses. If applicable, describe which groupings were chosen, and why			Page 8
Statistical methods	12	(a) Describe all statistical methods, including those used to control for confounding<br>(b) Describe any methods used to examine subgroups and interactions<br>(c) Explain how missing data were addressed<br>(d) *Cohort study* - If applicable, explain how loss to follow-up was addressed<br>*Case-control study* - If applicable, explain how matching of cases and controls was addressed<br>*Cross-sectional study* - If applicable, describe analytical methods taking account of sampling strategy<br>(e) Describe any sensitivity analyses			Page 8<br>N/A<br>N/A<br>Page 8<br>N/A
Data access and cleaning methods		..		RECORD 12.1: Authors should describe the extent to which the investigators had access to the database population used to create the study population.	Please see reference 8

For peer review only

				RECORD 12.2: Authors should provide information on the data cleaning methods used in the study.	Please see reference 8
Linkage		..		RECORD 12.3: State whether the study included person-level, institutional-level, or other data linkage across two or more databases. The methods of linkage and methods of linkage quality evaluation should be provided.	Please see reference 8
**Results**					
Participants	13	(a) Report the numbers of individuals at each stage of the study (*e.g.*, numbers potentially eligible, examined for eligibility, confirmed eligible, included in the study, completing follow-up, and analysed)<br>(b) Give reasons for non-participation at each stage.<br>(c) Consider use of a flow diagram		RECORD 13.1: Describe in detail the selection of the persons included in the study (*i.e.,* study population selection) including filtering based on data quality, data availability and linkage. The selection of included persons can be described in the text and/or by means of the study flow diagram.	Figure 1
Descriptive data	14	(a) Give characteristics of study participants (*e.g.*, demographic, clinical, social) and information on exposures and potential confounders<br>(b) Indicate the number of participants with missing data for each variable of interest<br>(c) *Cohort study* - summarise follow-up time (*e.g.*, average and total amount)			Table 1
Outcome data	15	*Cohort study* - Report numbers of outcome events or summary measures over time<br>*Case-control study* - Report numbers in each exposure			Figures 2 and 3

For peer review only

		category, or summary measures of exposure<br>*Cross-sectional study* - Report numbers of outcome events or summary measures			
Main results	16	(a) Give unadjusted estimates and, if applicable, confounder-adjusted estimates and their precision (e.g., 95% confidence interval). Make clear which confounders were adjusted for and why they were included<br>(b) Report category boundaries when continuous variables were categorized<br>(c) If relevant, consider translating estimates of relative risk into absolute risk for a meaningful time period			Page 8-9
Other analyses	17	Report other analyses done—e.g., analyses of subgroups and interactions, and sensitivity analyses			N/A
**Discussion**					
Key results	18	Summarise key results with reference to study objectives			
Limitations	19	Discuss limitations of the study, taking into account sources of potential bias or imprecision. Discuss both direction and magnitude of any potential bias		RECORD 19.1: Discuss the implications of using data that were not created or collected to answer the specific research question(s). Include discussion of misclassification bias, unmeasured confounding, missing data, and changing eligibility over time, as they pertain to the study being reported.	Page 9
Interpretation	20	Give a cautious overall interpretation of results considering objectives,			Page 9-11

For peer review only

		limitations, multiplicity of analyses, results from similar studies, and other relevant evidence			
Generalisability	21	Discuss the generalisability (external validity) of the study results			Page 9-11
**Other Information**					
Funding	22	Give the source of funding and the role of the funders for the present study and, if applicable, for the original study on which the present article is based			Page 11
Accessibility of protocol, raw data, and programming code		..		RECORD 22.1: Authors should provide information on how to access any supplemental information such as the study protocol, raw data, or programming code.	Page 12

*Reference: Benchimol EI, Smeeth L, Guttmann A, Harron K, Moher D, Petersen I, Sørensen HT, von Elm E, Langan SM, the RECORD Working Committee. The REporting of studies Conducted using Observational Routinely-collected health Data (RECORD) Statement. *PLoS Medicine* 2015; in press.

*Checklist is protected under Creative Commons Attribution (CC BY) license.

For peer review only
